# Nerve injury disrupts temporal processing in the spinal cord dorsal horn through alterations in PV^+^ interneurons

**DOI:** 10.1016/j.celrep.2024.113718

**Published:** 2024-01-30

**Authors:** Genelle Rankin, Anda M. Chirila, Alan J. Emanuel, Zihe Zhang, Clifford J. Woolf, Jan Drugowitsch, David D. Ginty

**Affiliations:** 1 Department of Neurobiology, Harvard Medical School, Boston, MA 02115, USA; 2 Howard Hughes Medical Institute, Harvard Medical School, Boston, MA 02115, USA; 3 F.M. Kirby Neurobiology Center, Boston Children’s Hospital, Boston, MA 02115, USA; 4 Lead contact

## Abstract

How mechanical allodynia following nerve injury is encoded in patterns of neural activity in the spinal cord dorsal horn (DH) remains incompletely understood. We address this in mice using the spared nerve injury model of neuropathic pain and *in vivo* electrophysiological recordings. Surprisingly, despite dramatic behavioral over-reactivity to mechanical stimuli following nerve injury, an overall increase in sensitivity or reactivity of DH neurons is not observed. We do, however, observe a marked decrease in correlated neural firing patterns, including the synchrony of mechanical stimulus-evoked firing, across the DH. Alterations in DH temporal firing patterns are recapitulated by silencing DH parvalbumin^+^ (PV^+^) interneurons, previously implicated in mechanical allodynia, as are allodynic pain-like behaviors. These findings reveal decorrelated DH network activity, driven by alterations in PV^+^ interneurons, as a prominent feature of neuropathic pain and suggest restoration of proper temporal activity as a potential therapeutic strategy to treat chronic neuropathic pain.

## In brief

Rankin et al. have identified reduced spike timing synchrony and correlated activity in spinal cord dorsal horn interneurons in a peripheral nerve injury model of neuropathic pain. Diminished reactivity of PV^+^ dorsal horn interneurons following nerve injury is responsible for the decorrelated spinal cord dorsal horn network activity.

## Graphical Abstract

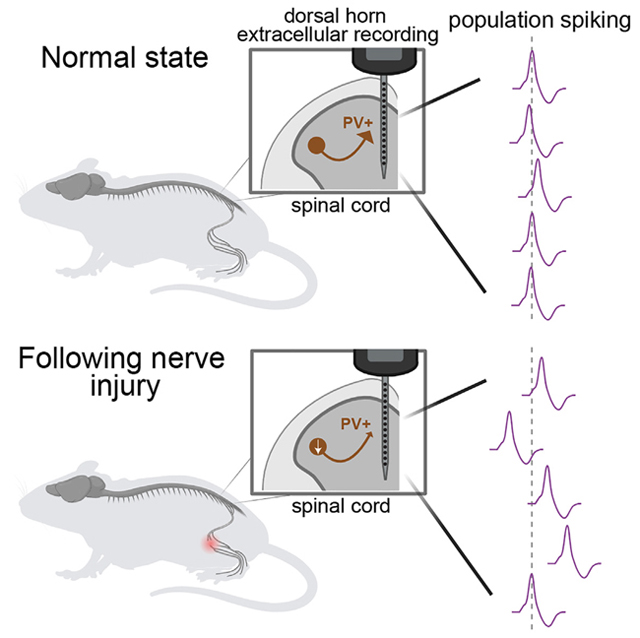

## INTRODUCTION

An understanding of how the somatosensory system enables perception and reactivity to mechanical stimuli acting on the skin will guide development of treatments for disorders of touch over-reactivity and mechanical pain. Chronic neuropathic pain, which can result from injury to the nervous system and is accompanied by painful reactivity to normally innocuous touch, called mechanical allodynia, afflicts between 3% and 17% of the global population,^1–3^ and current therapies offer only moderate symptom amelioration and are associated with deleterious side effects.^4,5^ Defining the neurophysiological basis of mechanical allodynia has been a challenge, and previous findings related to the induction and expression of this form of painful touch have suggested sites of dysfunction in peripheral sensory neurons, the spinal cord, and the brain.^1,6–20^

The first stage of integration of tactile signals flowing from the periphery is the spinal cord dorsal horn (DH). In the DH, axons of primary sensory neurons that innervate the skin and convey discrete streams of sensory information, including those in response to innocuous and noxious touch, synapse onto functionally diverse populations of interneurons as well as small populations of projection neurons.^21–28^ The mechanosensory DH contains 10 or more interneuron subtypes, characterized based on morphological, intrinsic physiological, molecular, and synaptic properties, at least four of which are inhibitory interneurons that collectively constitute ~30% of the DH neuronal population.^28–36^ DH inhibitory interneurons mediate two principal forms of spinal inhibition: feedback inhibition via axo-axonic synapses onto primary afferent terminals, also known as presynaptic inhibition (PSI), and feedforward inhibition (FFI) through axo-dendritic and axo-somatic synapses onto other interneurons and projection neurons.^28,30,37,38^ Spinal cord PSI and FFI are both necessary for normal output from the DH via projection neurons, including those projecting to higher-order brain regions that underlie tactile perception and associated behavioral responses.^21,39–41^

Changes in spontaneous and evoked activity of DH interneurons, particularly alterations in inhibitory interneurons leading to disinhibition, are a potential underlying cause of mechanical allodynia in neuropathic pain states,^25,30,42–44^ and indeed, several DH interneuron subtypes have been implicated from morphological, behavioral, and *in vitro* physiological analyses.^45–50^ However, the functional consequences of altered DH interneurons for population circuit dynamics of the intact spinal cord are not understood. Thus, while synaptic inhibition can shape sensitivity, sensory tuning, spike timing, and network dynamics in other regions of the nervous system, including the cortex,^51–56^ how alterations of synaptic inhibition in the DH in neuropathic pain states influence DH network activity, and the mechanisms and functional consequences, are unknown. Here, using in vivo multielectrode array (MEA) recordings, we report that DH interneurons exhibit temporal disorganization of spike patterns, but not hypersensitivity, in the injury-induced peripheral neuropathic pain state. Our findings also show that nerve injury and mechanical allodynia are associated with reduced parvalbumin^+^ (PV^+^) interneuron activity, and that inhibition of these interneurons in uninjured mice both recapitulates decorrelated and desynchronized activity across the DH and causes concomitant pain-like behavior. Interestingly, alterations of other spinal cord inhibitory motifs, including disruption of GABA_A_R (GABA_A_ receptor)-dependent PSI, which has been theorized to underlie mechanical allodynia,^57–59^ results in increased sensitivity, hyper-correlated DH activity, and behavioral over-reactivity but not pain. Thus, a decrease in temporally correlated network activity in the DH, and not network over-reactivity, is a feature of the allodynic spinal cord, and restoration of proper network dynamics may be required to alleviate mechanical allodynia in chronic neuropathic pain states.

## RESULTS

### *In vivo* spinal cord interneuron tuning in a mouse model of neuropathic pain

**Figure 1.**
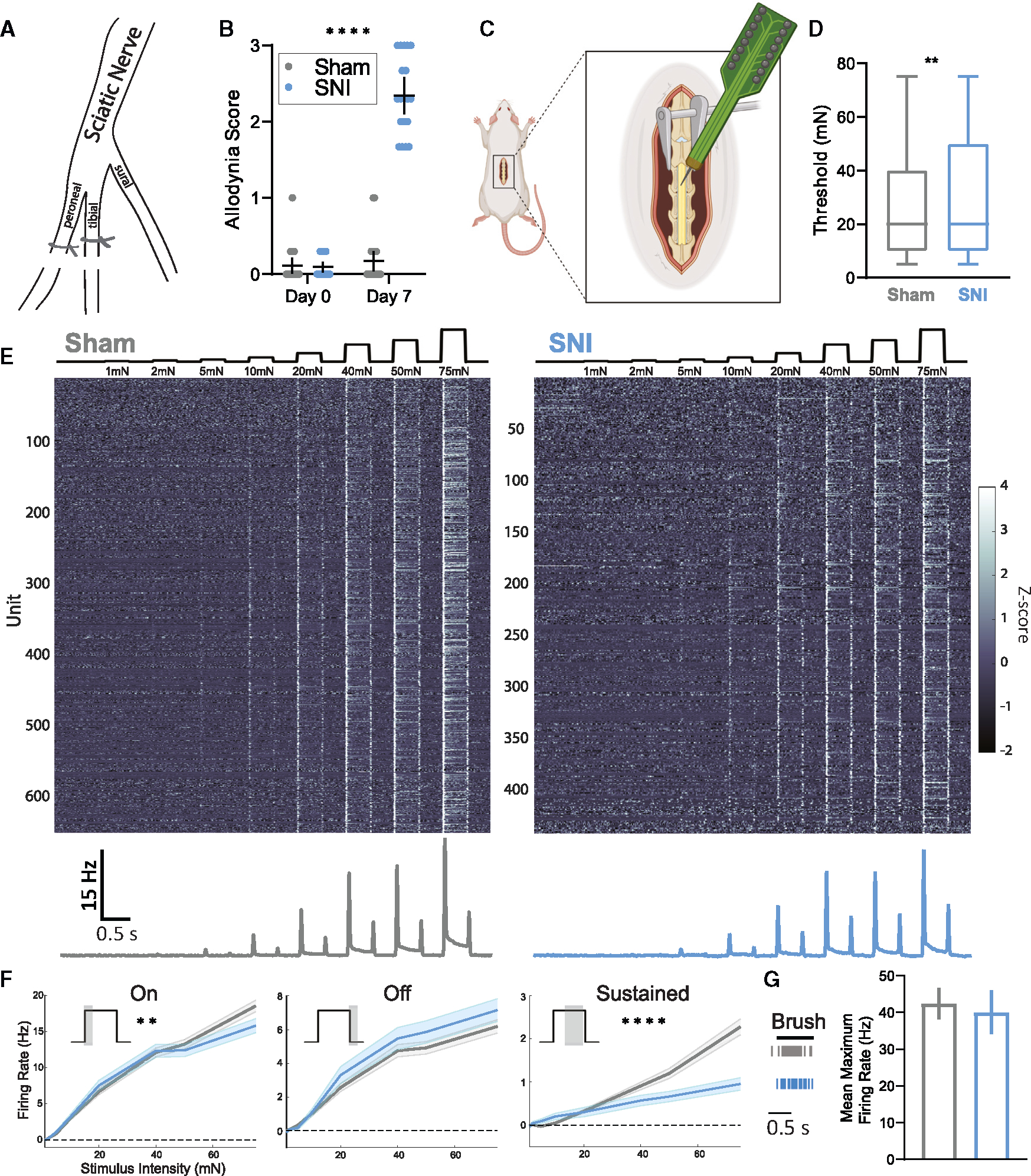
Mechanical allodynia following SNI is not associated with general physiologic over-reactivity across dorsal horn neurons (A) Diagram of the spared nerve injury model used to induce mechanical allodynia. The peroneal and tibial branches of the sciatic nerve are ligated and transected, sparing the sural branch, which innervates the lateral hindpaw. (B) Dynamic allodynia score compared at day 0 (prior to surgery) and day 7 (post surgery) between sham (N = 22) and SNI (N = 19) mice. Kruskal-Wallis H test with post hoc Dunn’s test (H[3, 82] = 51.37; p < 0.0001). SNI day 7 is significantly different from all other time points and conditions (****). (C) Diagram of *in vivo* spinal cord MEA experimental setup. Created with BioRender.com. (D) Distribution of indentation thresholds across DH neurons in sham (n = 642, gray) and SNI (n = 479, blue) mice. Mann-Whitney U test. (E) Indentation responses for DH units in sham and SNI conditions. Top: force traces aligned to the heatmaps of Z-scored firing rates for each condition. Bottom: mean baseline-subtracted firing rate peristimulus time histograms (PSTHs). (F) Average baseline-subtracted firing rates (±SEM) for DH units in sham and SNI groups at step indentation onset (on: 0–50 ms after step onset), offset (off: 0–50 ms after step offset), and sustained (sustained: 0–200 ms before step offset) periods. On: two-way ANOVA (F[1, 8,952] = 9.024, p = 0.0027). Sustained: two-way ANOVA (F[1, 8,952] = 36.72, p < 0.0001). (G) DH neurons responding to gentle brush strokes of the lateral hindpaw. Left: raster plot of an example sham (gray) and SNI (blue) neuron responding to brush. Right: average maximum brush evoked firing rates. Bars: mean. Error bars: 95% confidence interval (CI). Number of animals/cells (N/n). **p < 0.01, ****p < 0.0001. See Table S1 for statistical details.

To assess *in vivo* physiological alterations in DH circuitry following spared nerve injury^60^ (SNI; Figure 1A) and the development of mechanical allodynia (Figure 1B), we characterized the response properties of individual DH interneurons by recording activity from dozens of neurons simultaneously using *in vivo* MEAs.^21^ In urethane-anesthetized mice, the region of lumbar spinal cord where neuronal receptive fields of lateral hindpaw glabrous skin are concentrated was targeted for recordings (Figure 1C) because this skin region remains innervated by sural nerve peripheral neurons after SNI and exhibits behavioral over-reactivity to normally innocuous mechanical stimuli. Force-controlled steps of indentation ranging from 1 to 75 mN as well as gentle brush strokes were delivered to the lateral region of hindpaw glabrous skin. All recordings were done within 7–16 days post SNI surgery unless otherwise stated.

Using the dynamic brush assay,^48^ robust nocifensive behaviors, including paw withdrawal, lateral kicking, and paw licking, were observed in all mice subjected to SNI but not sham surgery (Figures 1B and S1A). Sham and SNI mice were then used to assess the sensitivity and response properties of DH interneurons to test the hypothesis that SNI would cause a general increase in DH interneuron reactivity to the mechanical stimuli that cause pain. For this electrophysiological analysis, 642 randomly recorded single units (Figures S1B and S1C) spanning lamina I through V from 22 sham mice were obtained and compared to 479 units obtained from 19 SNI mice. Interestingly, although SNI animals exhibited dramatically increased behavioral responsivity to light touch stimuli (Figure 1B), no overall physiological over-reactivity was observed across the population of DH interneurons (Figures 1D–1G). In fact, contrary to our expectation, on average, DH interneurons exhibited a slight increase in their response thresholds to steps of static indentation following SNI (Figure 1D). We also generated tuning curves to investigate possible changes in firing rates to various components of the indentation steps. This analysis revealed that, as a population, DH interneurons in SNI mice did not exhibit changes in evoked firing rates during the OFF component of step indentations, but they did show decreased firing rates during the sustained portion of indentation steps and a small reduction in firing during the ON component (Figures 1E and 1F). In addition, the same brush stimulus used in the dynamic brush behavioral assay was used to stimulate the identical lateral hindpaw region while DH interneuron activity was recorded. There was no change in the maximum evoked firing rate to the brush stimulus between sham and SNI mice (Figure 1G). Thus, although dramatic behavioral over-reactivity to gentle touch was observed after nerve injury, as a population, DH interneurons did not exhibit physiological over-reactivity to tactile stimuli.

### Temporal activity patterns are disorganized after nerve injury

**Figure 2.**
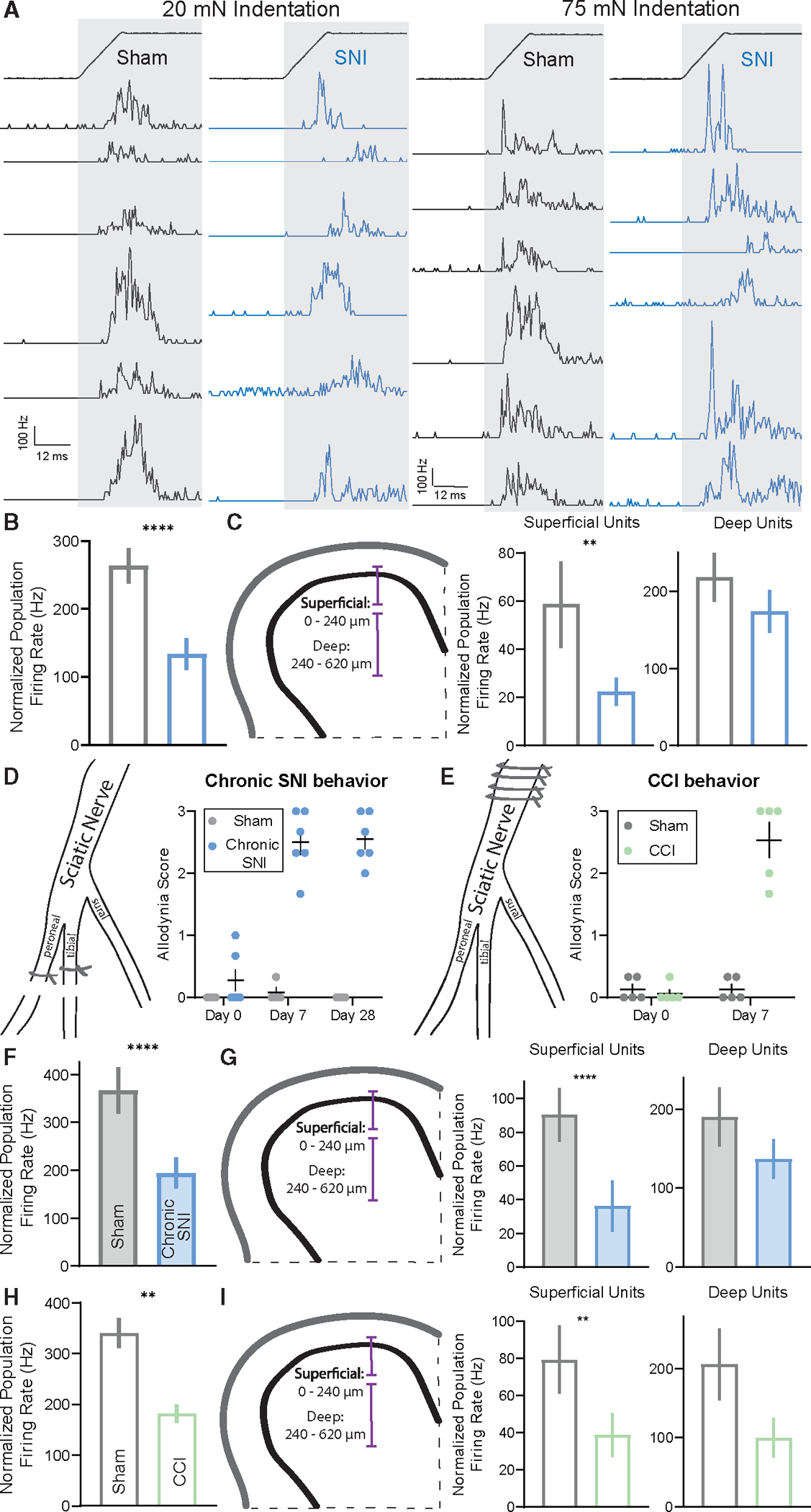
Temporal alignment of population level activity is altered in models of mechanical allodynia (A) PSTHs (0.5 ms bins) of simultaneously recorded units showing temporal alignment at indentation onset at 20 mN (left) and 75 mN (middle) in sham and SNI. (B) Population coupling quantified as normalized population firing rate. (C) Left: schematic of the DH subdivided into superficial and deep segments. Right: population coupling of superficial and deep units across conditions. (D) Diagram of the spared nerve injury model used to induce mechanical allodynia to test chronic allodynia state, followed by dynamic allodynia score compared at day 0 (prior to surgery), day 7 (post surgery), and day 28 (post surgery) between sham (N = 4) and SNI (N = 6) mice. One-way ANOVA with post hoc Tukey’s test (F[5, 24] = 64.28; p < 0.0001). SNI day 7 and day 28 were significantly different from all other time points and conditions (****). Error bars: SEM. (E) Diagram of the chronic constriction injury model used to induce mechanical allodynia. The sciatic nerve is ligated four times proximal to the branching of peroneal, tibial, and sural nerves (left). Right: dynamic allodynia score compared at day 0 (prior to surgery) and day 7 (post surgery) between sham (N = 5) and CCI (N = 5) mice. Kruskal-Wallis H test with post hoc Dunn’s test (H[3, 20] = 12.74; p < 0.0001). CCI day 7 is significantly different from all other time points and conditions (****). (F) As in (B), for chronic SNI model. (G) As in (C), for chronic SNI model. (H) As in (B), for CCI model. (I) As in (C), for CCI model. Note the p value comparing sham and CCI deep units is ~0.06. Bars: mean. Error bars: 95% CI. Number of animals/cells (N/n). Mann-Whitney U tests, *p < 0.05, **p < 0.01, ****p < 0.0001. See Table S1 for statistical details.

The lack of overall increased sensitivity and evoked activity in the DH of SNI mice led us to ask whether other aspects of DH circuit properties and firing patterns may be altered. The nature of our MEA recording configuration allowed for the activity of many neurons to be recorded simultaneously, and therefore, whether and how temporal activity patterns change across the DH as a population in SNI mice was determined. To assess spike timing precision across populations of DH neurons, a population coupling metric was used to determine the extent to which an individual DH neuron’s spiking is correlated with other neurons in the population.^61,62^ Thus, by analyzing spike patterns across all simultaneously recorded units with 1 ms time bins, the extent of synchronous population activity was determined during both indentation and brushing of the skin. A decrease in the synchrony of evoked spiking (see STAR Methods) during both indentation steps (Figures 2A and 2B) and brushing (Figure S2A) was observed in SNI mice compared to sham controls. To further assess the extent to which firing synchrony was altered after SNI, a range of bin sizes was used for the population coupling analysis. A deficit in synchronous firing was still observed when the bin size was expanded from 1 to 3 ms but not to 10 ms (Figure S2B), thus constraining the timescale of desynchrony in SNI animals to precise millisecond spike timing. This observed deficit in temporally precise evoked synchronous population activity was observed across periods of evoked activity but was most prominent during the onset and offset of indentations steps (Figure S2C). Also of note, we observed a small but significant increase in the latency to first spike for the ON component of step indentations at higher forces as well as an increase in the jitter of the ON response (Figure S2D) in SNI mice.

Because the superficial DH receives and processes information about high-threshold mechanical stimuli and noxious stimuli, as well as thermal and other stimuli, whereas the deep DH receives many inputs from primary sensory neurons that encode innocuous, light touch signals,^63–65^ we next asked whether the altered synchrony of evoked spiking in SNI animals is observed across both superficial and deeper regions or is more restricted. To test this, the DH was divided into superficial (units with recorded depths between 0 and ~240 μm in the spinal cord) and deep (units with depths between ~240 and 620 μm) regions, and synchronous population activity was calculated for units within these boundaries. After SNI, synchronous population activity within deep DH units was comparable to sham controls; however, synchronous activity in the superficial DH was reduced by more than half (Figure 2C).

Disorganized population coupling across DH units was not observed immediately after nerve injury (4 h post surgery), which is prior to the development of mechanical allodynia (Figures S2E and S2F), suggesting temporal disorganization is concomitant with pain behaviors. To test for similar changes in network activity across allodynic states, we expanded our allodynia models to include a later stage SNI time point to monitor chronic pain (chronic SNI), as well as the chronic constriction injury (CCI) model of mechanical allodynia. Both chronic SNI and CCI mice displayed allodynic behaviors 7 days after their respective surgeries (Figures 2D, 2E, S3A, and S3B), decreased evoked firing, and increased response thresholds (Figures S3C–S3F). Comparable to the SNI model, disorganized population coupling was observed in both chronic SNI and CCI mice (Figures 2F–2I, S3G, and S3H), with the strongest deficit observed in superficial DH synchronous population activity (Figures 2G and 2I).

Together, deficits in population coupling were observed in both early (7–16 days post SNI and CCI surgery) and later stages (28–35 days post SNI surgery) of the neuropathic pain state, suggesting that disorganization of temporal firing patterns in the DH emerges at the same time as the behavioral pain response and persists throughout the transition from acute to chronic pain. Thus, a temporal disorganization of spiking in the DH, but not overall physiological hypersensitivity, occurs coincidently with the development of behavioral over-reactivity to tactile stimuli.

**Figure 3.**
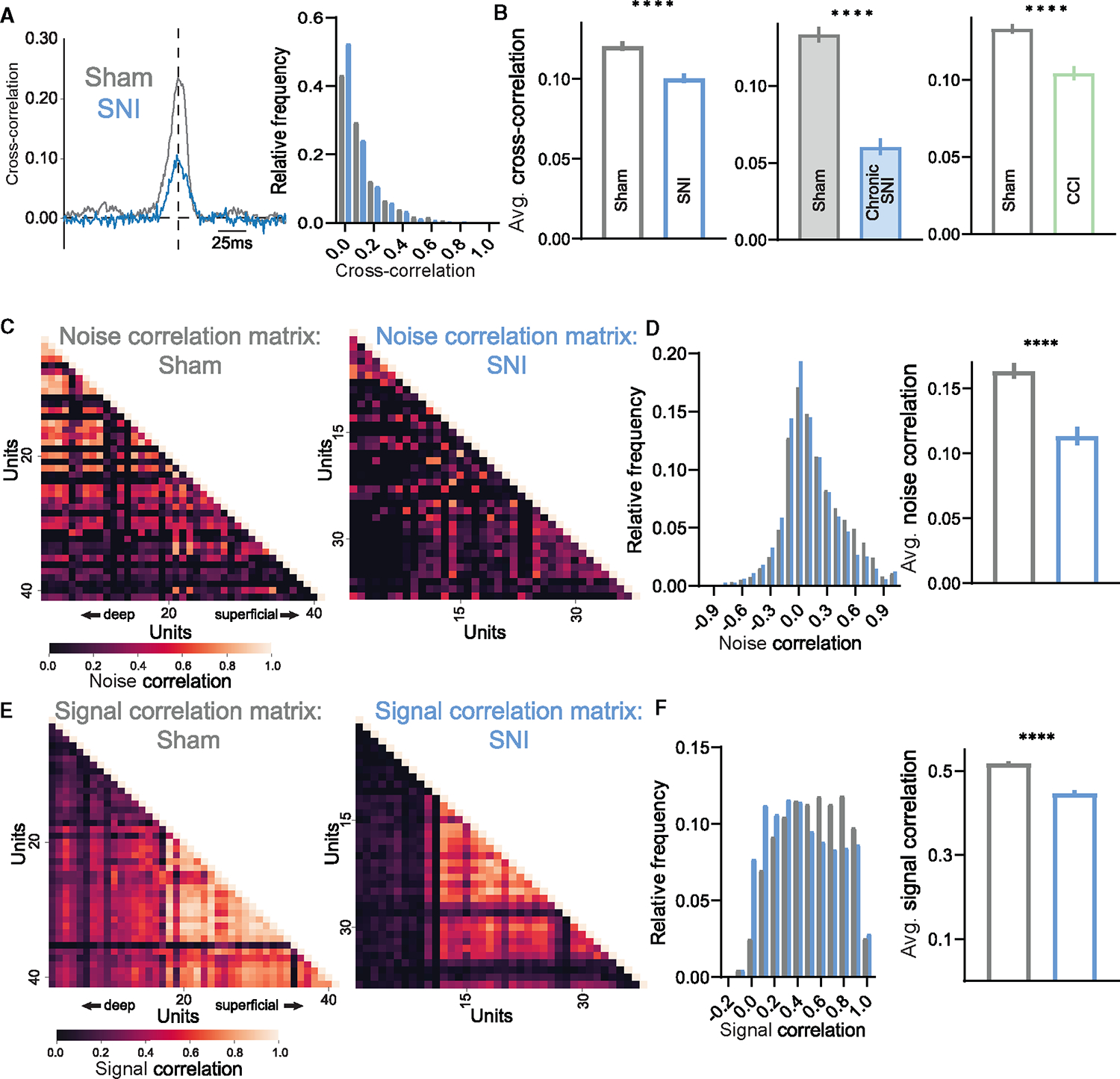
Neuronal firing correlations are decreased in the allodynic dorsal horn (A) Example cross-correlogram of one sham (gray) and SNI (blue) interneuron pair (left) followed by distribution of synchrony cross-correlations for pairs of DH neurons (at time lag = 0). (B) Average paired synchrony cross-correlations across SNI (left), chronic SNI (middle), and CCI (right) models of mechanical allodynia throughout indentation steps. (C) Example noise correlation matrices of simultaneously recorded units in sham and SNI mice. (D) Distribution of noise correlations for pairs of DH neurons, followed by average noise correlations. Note the increase of SNI neuron pairs with correlation coefficients in bins −0.3 to 0.0 and decrease in bins 0.5 to 0.8. (E) Example signal correlation matrices of simultaneously recorded units in sham and SNI mice. (F) Distribution of signal correlations for pairs of DH neurons, followed by average signal correlations. Note the increase of SNI neuron pairs with correlation coefficients in bins 0.0 to 0.3 and decrease in bins 0.5 to 0.8. Bars: mean. Error bars: 95% CI. Mann-Whitney U tests, *p < 0.05, **p < 0.01, ****p < 0.0001. See Table S1 for statistical details.

When calculating firing synchrony between pairs of neurons using spike cross-correlograms^66^ and computing the correlation at time lag 0, similar deficits in synchronous activity were observed across SNI, chronic SNI, and CCI groups (Figures 3A and 3B). Although pairs of DH neurons in all groups showed an overall decrease in paired synchronous firing (Figure 3B), and specifically deficits during periods of indentation onset, only the SNI condition showed decreases across all three 50 ms sampled periods of evoked activity (onset, offset, and sustained; Figures S4A–S4C). This suggests the precise synchrony of touch-evoked firing in DH neurons is compromised in neuropathic pain states; however, the nature and extent of disruption may vary slightly across models.

As a complement to measuring spike timing correlations across millisecond timescales, longer timescale activity correlations (across tens of milliseconds to seconds) were examined to better understand network tuning and connectivity. Through spike count correlations during windows of spontaneous activity,^66^ trial-to-trial variability between pairs of neurons (i.e., noise correlations) was assessed. SNI and chronic SNI mice showed reduced noise correlations compared to sham controls (Figures 3C, 3D, and S4D). While CCI mice had increased noise correlations (Figure S4F), all three allodynic groups also displayed smaller pairwise signal correlations,^66^ a measurement of tuning similarity, to both indentation and stroke of the hindpaw (Figures 3E, 3F, and S4D–S4F). To look at potential changes in large-scale neuronal oscillations in neuropathic mice, local field potentials (LFPs) across the timescale of seconds were recorded, and the power spectral density was computed. No differences in the power across frequencies were observed between sham and SNI mice (Figure S4G), which is not surprising when considering that most temporal misalignment occurs within 10 ms time periods (Figure S2B). Together, the decreases in noise and signal correlations in SNI models suggest that pairs of DH interneurons share fewer common inputs and are less similarly tuned to indentation steps and brush strokes.

### PV^+^ interneuron activity is altered in mechanical allodynia

Previous studies of cortical circuitry have implicated fast-spiking inhibitory interneurons in the control of spike timing, neuronal tuning, and firing correlations.^51^ Additionally, a deficit of DH inhibition has been proposed to underlie the development of neuropathic pain.^25,42–50,57^ These prior findings led us to hypothesize that dysfunction of DH fast-spiking inhibitory interneurons may lead to the alterations in temporal processing observed in mice with mechanical allodynia. Using the SNI model of mechanical allodynia to investigate possible abnormalities in DH inhibition following nerve injury, we first analyzed extracellular action potential waveforms to dissect narrow versus broad spiking waveforms, as is routinely done in cortical datasets, to identify putative inhibitory and excitatory neurons.^67^ As opposed to the cortex, where both narrow and broad waveforms are readily observed (Figure S1D),^68,69^ DH interneuron waveforms^21^ are more uniform and cannot be easily subdivided (Figure S1E). Using this extracellular waveform analysis, we observed no gross changes in DH waveforms between sham and SNI conditions (Figures S1F–S1I). We therefore turned to opto-genetic approaches to identify inhibitory interneurons and genetically distinct interneuron populations. All inhibitory interneurons were targeted using *Vgat*^*iCre*^*; R26*^*LSL-ChR2-YFP*^ mice for identification using optical stimuli, and their responses in the spinal cord to mechanical stimuli were recorded *in vivo* using MEAs (Figures S5A–S5C). As a population, Vgat^+^ interneurons in SNI mice did not exhibit changes in response thresholds or evoked and spontaneous firing rates compared to sham controls (Figures S5D–S5G).

**Figure 4.**
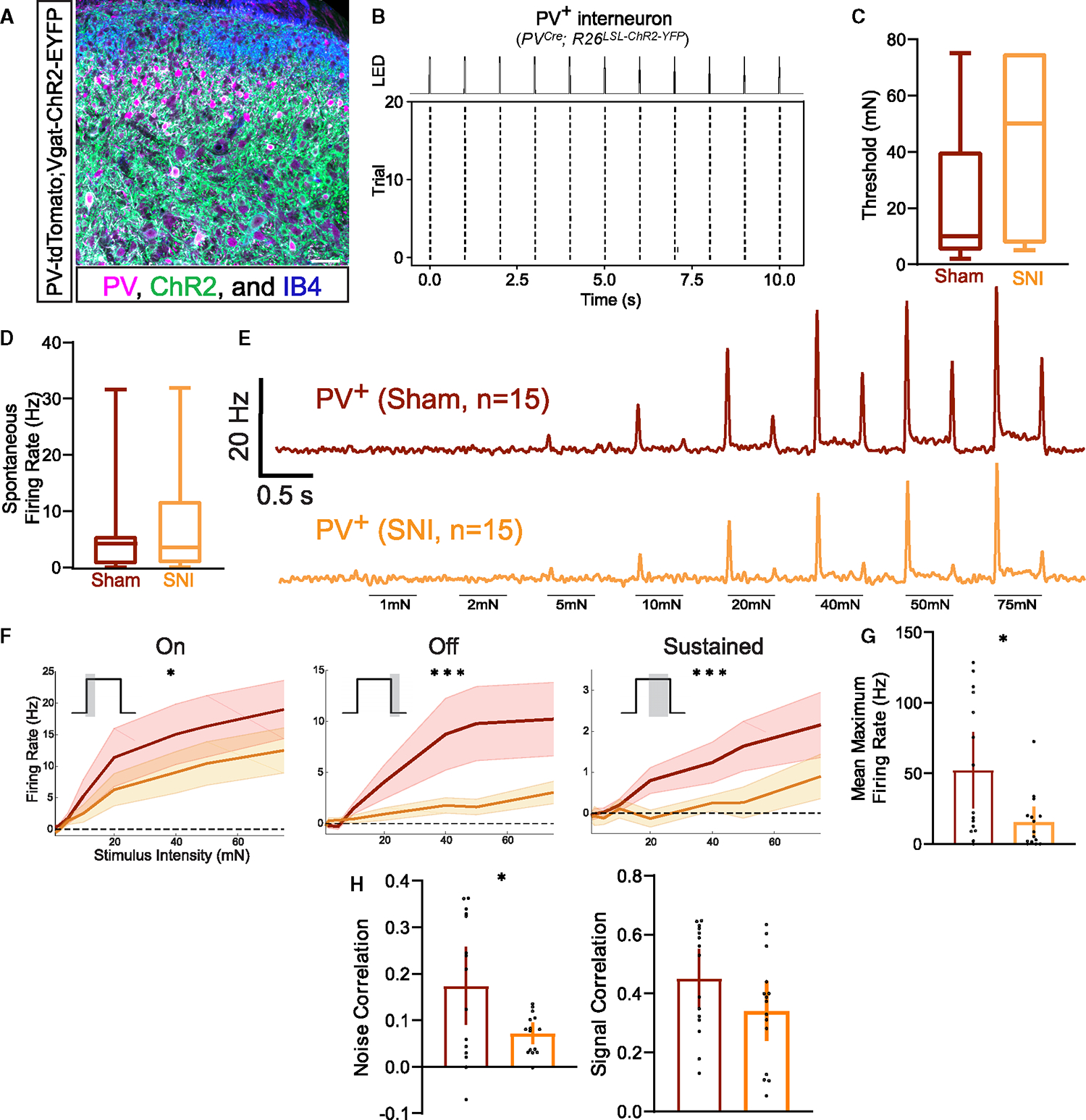
Dorsal horn PV^+^ interneuron activity is decreased in mice following SNI (A) Transverse spinal cord section showing PV^+^ interneurons in comparison to all Vgat^+^ cells (PV-tdTomato; Vgat-ChR2-EYFP mouse). PV^+^ neurons, magenta; Vgat-ChR2^+^ neurons, green; IB_4_ binding, blue (labels lamina IIi). Scale bar: 50 μm. (B) Raster plot of light-evoked spikes in a PV^+^ interneuron (*PV*^*Cre*^*; R26*^*LSL-ChR2-YFP*^). (C) Indentation thresholds across PV^+^ neurons in sham (N = 3, n = 15) and SNI (N = 3, n = 15) mice. (D) Spontaneous firing rates of PV^+^ interneurons in sham (N = 3, n = 15) and SNI (N = 3, n = 15) mice. (E) Mean baseline-subtracted firing rate PSTHs for sham and SNI PV^+^ neurons. (F) Average baseline-subtracted firing rates (±SEM) for PV^+^ neurons in sham and SNI groups at step indentation on, off, and sustained periods. On: two-way ANOVA (F[1, 240] = 5.602 p = 0.0187). Off: two-way ANOVA (F[1, 240] = 14.59, p = 0.0002). Sustained: two-way ANOVA (F[1, 240] = 11.41, p = 0.0009). (G) Sham and SNI PV^+^ interneurons average maximum brush evoked firing rates. Unpaired t test. (H) Noise (left) and indentation signal (right) correlations between PV^+^ interneurons and neighboring cells in sham and SNI mice. Unpaired t test. Bars: mean. Error bars: 95% CI. Number of animals/cells (N/n). *p < 0.05, **p < 0.01, ***p < 0.001. See Table S1 for statistical details.

Since *Vgat*^*iCre*^ labels all inhibitory interneurons, it remained possible that a smaller subset of inhibitory neurons may be altered after SNI, and these changes could be masked when analyzing the broader inhibitory interneuron population. Because of the net population reduction in sustained responses after SNI, we suspected that a fast-spiking inhibitory interneuron subtype known to have strong sustained responses to mechanical stimuli, the PV^+^ inhibitory interneurons of the DH,^21^ may be affected in SNI mice. This population is of interest because *in vitro* and behavioral studies have suggested that PV^+^ interneuron intrinsic excitability is altered after SNI, and that PV^+^ interneuron output and connectivity are necessary for behavioral over-reactivity to mechanical stimuli.^45,50,70^ Although these prior findings implicated PV^+^ interneurons in neuropathic pain, whether evoked activity and sensory tuning of PV^+^ neurons change *in vivo* in neuropathic pain states remains unclear. Therefore, we opto-tagged PV^+^ interneurons using *PV*^*Cre*^*; R26*^*LSL-ChR2-YFP*^ mice for *in vivo* electrophysiological recordings to assess their physiological responses to tactile stimuli following SNI (Figure 4B). It is noteworthy that *PV*^*Cre*^ labels subsets of excitatory and inhibitory neurons; up to 66%–95% of DH PV^+^ cells are co-labeled with inhibitory markers^28,32,50^ (Figure 4A). After SNI, PV^+^ interneurons did not exhibit changes in their response thresholds or spontaneous firing rates (Figures 4C and 4D). However, these interneurons displayed decreased firing to the sustained and OFF portions of step indentations, as well as reduced firing to brushing of the skin (Figures 4E–4G). PV^+^ interneurons also showed decreased noise correlations with other simultaneously recorded units suggesting a reduction in PV^+^ interneuron connectivity after SNI (Figure 4H), consistent with a previous anatomical study,^50^ while no changes in signal correlations were observed (Figure 4H).

In a complementary set of experiments, CCK^+^ interneurons, a broad population of excitatory neurons with distinct evoked firing responses compared to inhibitory PV^+^ interneurons, implicated in neuropathic pain via their connections with corticospinal tract axons,^71^ were also examined after SNI using *CCK*^*iCre*^*;R26*^*LSL-ChR2-YFP*^ mice. These excitatory CCK^+^ interneurons showed increased responsivity at the OFF portion of the step indentations (Figures S5H–S5K), suggesting CCK^+^ interneurons may contribute to circuit disruption following nerve injury. This increased firing is consistent with a decrease in PV-mediated inhibition, since PV^+^ interneurons, which show reduced evoked firing following nerve injury (Figure 4F), form synapses onto at least some CCK^+^ interneurons.^50^

### PV^+^ interneurons control temporal activity patterns across spinal cord neurons and mechanical allodynia following nerve injury

**Figure 5.**
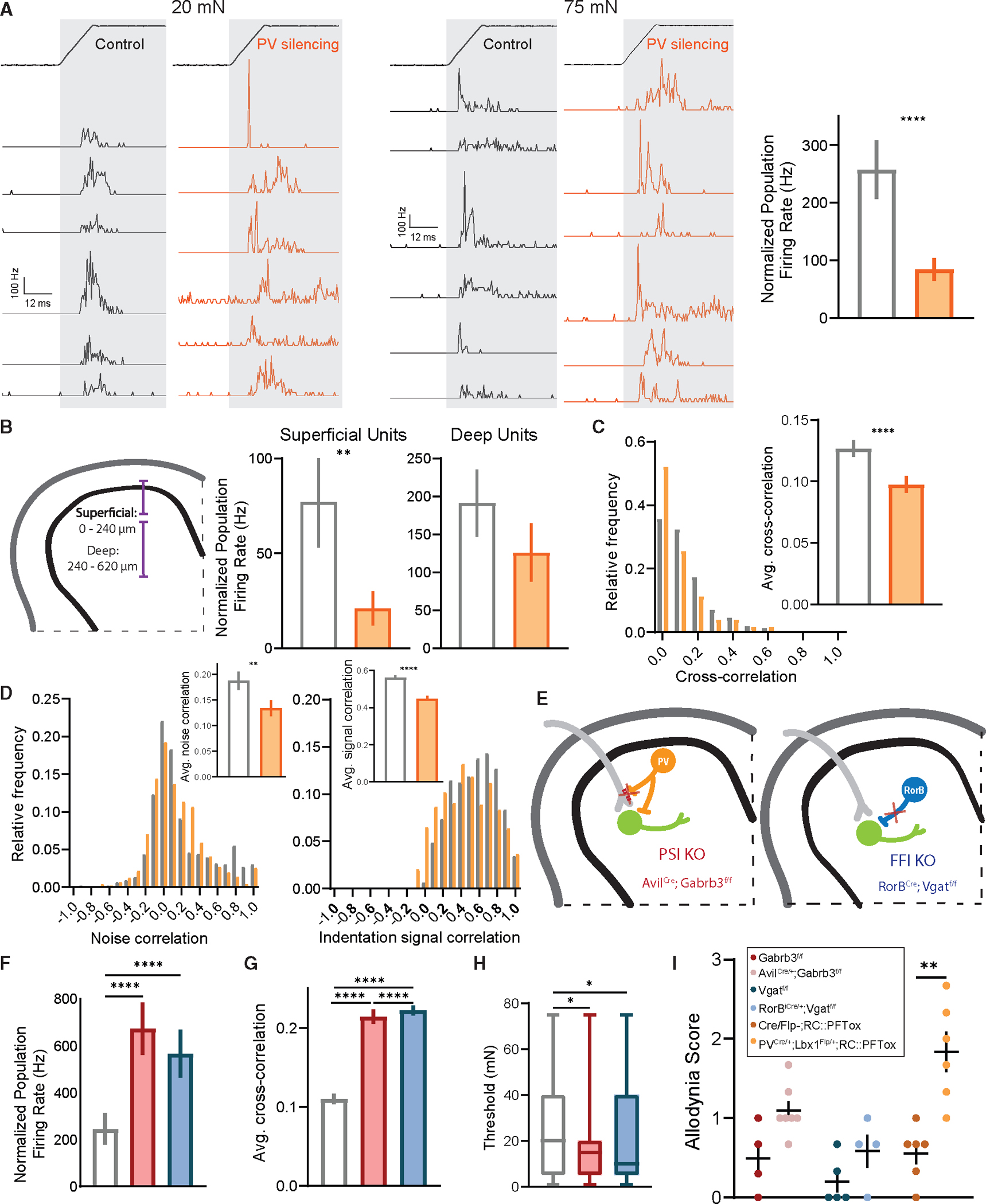
Deficits in temporally correlated activity across the DH and allodynia-like behavior after silencing PV^+^ interneurons (A) PSTHs (0.5 ms bins) of simultaneously recorded units showing temporal alignment at indentation onset at 20 mN (left) and 75 mN (middle) in control (*PV*^*Cre*^*;RC::PFtox*; N = 4) and PV-silencing (*PV*^*Cre*^*;Lbx1*^*FlpO*^*;RC::PFtox*; N = 4) conditions, followed by population coupling quantified as normalized population firing rate (right). Mann-Whitney U test. (B) Left: schematic of superficial and deep DH segments. Right: population coupling of superficial and deep units across conditions. Mann-Whitney U tests. (C) Distribution of synchrony cross-correlations for pairs of DH neurons in controls (white bars) and mutants (orange bars). Inset: average paired cross-correlations. Mann-Whitney U test. (D) Distributions of noise (left) and signal (right) correlations for pairs of DH neurons. Insets: average noise and signal correlations in controls (white bars) and mutants (orange bars). Mann-Whitney U tests. (E) Diagram of genetic strategies to silence all presynaptic DH inhibition (*Avil*^*Cre*^*;Gabrb3*^*f/f*^; PSI KO) and Rorβ-mediated feedforward inhibition (*Rorβ*^*iCre*^*;Vgat*^*f/f*^; FFI KO). (F) Population coupling for each condition. Kruskal-Wallis H test with post hoc Dunn’s test (H[2, 314] = 42.30; p < 0.0001). (G) Synchrony cross-correlations for neuron pairs. Kruskal-Wallis H test with post hoc Dunn’s test (H[2, 6,099] = 757.3; p < 0.0001). (H) Distribution of indentation thresholds between controls (*Vgat*^*f/f*^ or *Gabrb3*^*f/f*^, white, N = 3), PSI KOs (red, N = 3), and FFI KOs (blue, N = 3). One-way ANOVA with post hoc Tukey’s test (F[2, 311] = 4.606; p = 0.0107). (I) Dynamic allodynia score compared at baseline (no surgery). Mean and ±SEM plotted. Mann-Whitney U test. Bars: mean. Error bars: 95% CI. Number of animals (N). **p < 0.01, ***p < 0.001, ****p < 0.0001. See Table S1 for experimental details.

The marked reduction in PV^+^ interneuron firing following SNI raised the possibility that PV^+^ interneurons normally control DH temporal dynamics. In fact, in the cortex, inhibitory PV^+^ cells have been shown to regulate both precise spike timing and longer-scale neural oscillations, in particular gamma oscillations. We observed no changes in the relative power of gamma oscillations measured using LFPs in the DH in sham or SNI mice, nor did we see optogenetic activation of PV^+^ neurons increase gamma power, as observed in cortex^53,72^ (Figure S6A–S6C). To assess whether DH PV^+^ neurons regulate precise spike timing, PV^+^ interneurons were silenced with tetanus toxin using *PV*^*Cre*^*;Lbx1*^*FlpO*^*;RC::PFtox* mice,^73^ and spike timing precision with population coupling and paired firing synchrony was measured using *in vivo* MEA recordings. Consistent with a large population of mainly inhibitory neurons being silenced, increased firing rates were observed across DH neurons following PV^+^ interneuron silencing compared to controls (Figure S6D), confirming silencing efficacy. Remarkably, as observed in mice following nerve injury, evoked population coupling to both indentation and brush (Figures 5A, S6E, and S6F) was markedly decreased in PV-silenced mice. Moreover, as in SNI and CCI mice, these coupling deficits were most pronounced in the superficial DH (Figure 5B). Also similar to findings with SNI mice, the deficits in population coupling in PV-silenced mice diminished as bin size increased, thus resolving the synchrony deficits to millisecond spike timing (Figure S6G). Thresholds of DH interneurons remained unchanged in response to PV silencing (Figure S6H); however, as in SNI, paired synchronous firing and noise and signal correlations were decreased in PV-silenced mice (Figures 5C, 5D, S6I, and S6J), suggesting that DH interneurons share fewer common inputs, and that evoked responses to mechanical stimuli are more diverse when PV^+^-mediated synaptic transmission is compromised. No changes were observed in relative gamma power between control and PV-silenced mice LFPs (Figure S6K). Thus, PV^+^ interneurons exhibit reduced activity under SNI conditions, and silencing PV^+^ interneurons recapitulates the marked overall disorganization of temporal firing patterns observed in the neuropathic pain state, with specific deficits in the superficial DH (Figures S7A–S7C).

We next asked how other DH interneuron circuits contribute to synchrony in DH firing patterns and whether disrupting these circuits leads to similar temporal disorganization following nerve injury and PV^+^ interneuron silencing. Changes in DH inhibition have been broadly theorized to underlie the development of mechanical allodynia, and therefore, we manipulated two other inhibitory circuits, GABA_A_R-dependent PSI of primary afferent terminals^28,45,74^ via axo-axonic synapses and FFI driven by Rorβ inhibitory interneurons via axo-dendritic synapses^21^ (Figure 5E). To determine whether silencing GABA_A_R-dependent PSI results in coordinated activity deficits, and if it potentially underlies the coordinated activity changes following nerve injury, DH neuron activity was recorded in mice lacking GABA_A_ receptors on primary afferents, which are necessary for GABA_A_R-dependent PSI^40,41^ (*Avil*^*Cre*^*;Gabrb3*^*f/f*^ mice, Figure 5E). Using the same set of coordinated activity metrics, we observed increased population coupling, paired synchronous firing, and signal correlations (Figures 5F, 5G, and S7D) as well as decreased indentation thresholds (Figure 5H) in *Avil*^*Cre*^*;Gabrb3*^*f/f*^ mice, precisely the opposite of that observed following nerve injury or PV^+^ interneuron silencing. These findings indicate that ablating GABA_A_R-dependent PSI alone does not recapitulate the deficit in temporal correlations observed following PV^+^ interneuron silencing and SNI. We next used *Rorβ*^*iCre*^*;Vgat*^*f/f*^ mice, in which Rorβ inhibitory interneurons are silenced, to ask whether disrupting a non-PV^+^ interneuron contributing to FFI causes similar temporal changes across the DH (Figure 5E). Rorβ interneurons make axo-dendritic synapses in the deep DH (laminae IIiv–IV)^28^ and provide the majority of mechanically evoked FFI of post-synaptic dorsal column projection neurons.^21^ However, they also contribute to PSI in the superficial DH.^75^ These experiments using *Rorβ*^*iCre*^*;Vgat*^*f/f*^ mice revealed increases in sensitivity and correlated evoked activity (Figures 5F–5H and S7D) similar to the *Avil*^*Cre*^*;Gabrb3*^*f/f*^ mutant mouse PSI disruption model but distinct from the temporal dysregulation observed following SNI and PV^+^ interneuron silencing. Note that evoked firing increases in both *Avil*^*Cre*^*;Gabrb3*^*f/f*^ and *Rorβ*^*iCre*^*;Vgat*^*f/f*^ mice is consistent with silencing inhibitory circuits (Figure S7E).

The requirement of PV^+^ interneuron signaling for normal temporal patterns of activity in the superficial DH, in conjunction with our findings that both PV^+^ interneuron activity and correlated activity are markedly reduced in SNI, led us to explore the extent to which silencing PV^+^ interneurons, disrupting GABA_A_R-dependent PSI, or disrupting Rorβ-mediated FFI leads to mechanical allodynia as observed in nerve injury models. Thus, using the dynamic brush assay to test for mechanical allodynia, PV interneuron-silenced, GABA_A_R-dependent PSI-disrupted, and Rorβ interneuron-mediated FFI-disrupted mice were evaluated. PV^+^ interneuron-silenced mice exhibited allodynia that was comparable to that observed in SNI animals (Figures 5I and S7G). This finding is consistent with prior results showing that ablating PV^+^ interneurons increased punctate mechanical sensitivity.^50,70^ On the other hand, disruption of either GABA_A_R-dependent PSI or Rorβ-mediated FFI, both of which lead to increased physiological reactivity to step indentations^21^ and increased correlated activity in the DH (Figures 5F–5H and S7D), caused behavioral over-reactivity to light touch, based on paw withdrawal following the stimulation, but these manipulations did not cause pain-like behaviors as measured by lateral kicking and licking of the contacted paw (Figures 5I and S7G).^21,39–41,76^ It is worth noting that although the silencing strategies used above are developmental in nature, others have used adult silencing strategies to inhibit PV^+^ interneurons and observed similar behavior effects.^21,40,50^ Taken together, these findings suggest that decorrelated activity at the population level in the DH, resulting from altered PV^+^ interneuron activity, and not a generalized increase in evoked firing across the DH, underlies mechanical allodynia in a peripheral nerve injury-induced neuropathic pain state.

## DISCUSSION

Using *in vivo* multielectrode array electrophysiology, genetic labeling, and network-level activity analyses, we sought to identify physiological signatures that represent normal DH circuit function as well as circuit-level dysfunction underlying mechanical allodynia associated with neuropathic pain. Our findings suggest that a deficit in coordinated activity, including temporal misalignment of touch-evoked DH interneuron spiking, and not general over-reactivity to tactile stimuli, is a characteristic feature of the allodynic spinal cord. Additionally, we found that PV^+^ interneurons control rapid (millisecond timescale) temporal processing in the DH, and that reduced activity of these interneurons in the allodynic spinal cord is responsible for aberrant temporal processing of tactile signals. We propose that, following nerve injury, a reduction in DH PV^+^ fast-spiking interneuron activity underlies deficits in the synchrony of touch-evoked spiking in the DH, which produces mechanical allodynia.

### Decorrelated network activity, not generalized over-reactivity, in the dorsal horn is observed in a neuropathic pain model

After nerve injury, animals exhibit nocifensive behaviors in response to normally innocuous stimuli, and yet, our *in vivo* electrophysiological recordings showed a lack of increase in sensitivity or evoked firing across the general DH population. In fact, overall firing levels decreased during the sustained portion of step indentations in SNI animals. This suggests that the increased behavioral output is not directly linked to an overall increase in evoked spiking across the DH, as we had expected, and that other facets of neuronal processing are likely altered to account for the dramatic shift in behavioral reactivity. Indeed, we found that coordinated population activity and synchronous firing across DH neurons are markedly decreased after SNI and CCI. This change in synchronous population activity was not observed 4 h post nerve injury but only arose over days, coincident with the development of pain-like behavioral responses to light touch. It is worth noting that DH recordings were performed under urethane anesthesia, which can alter neuronal dynamics when compared to the unanesthetized state. Anesthesia was maintained throughout all tested conditions making all metrics comparable; however, future studies should address changes in DH activity in awake mice experiencing pain states.^77^

After nerve injury, individual interneurons of the DH are less coupled to the total population activity, compared to DH interneurons of control mice, meaning that there are more neurons that are “soloists,” less influenced by population-wide events compared to a predominance of “chorister” neurons observed in the control DH. Whether and how this loss of broad, synchronous population activity could lead to altered behavioral reactivity or perception of tactile stimuli are unclear. Synchrony is often proposed to enable efficient information transfer from one brain region to another. One possibility is that desynchronized responses in the DH result in an incomplete representation of tactile features in downstream brain regions and therefore disable the ability of the CNS to decode or match with internal predictions of sensory experiences, as posited by the theory of predictive coding.^78^ In fact, we observed a decrease in the similarity of neuronal tuning between pairs of neurons after nerve injury (decreased signal correlations). It is possible that divergent physiological DH interneuron response patterns could lead to “misinterpretation” of light touch stimuli as being noxious.

It is also possible that the observed disorganization of spike timing in the neuropathic pain state allows for signals to propagate to DH projection neurons that would normally be blocked or shunted by precisely timed inhibitory connections. Such unchecked signals arising from primary afferents or neighboring DH interneurons could result in altered sensitivity, evoked firing, latency to respond, and signal-to-noise ratios, both at the level of individual neurons and as a population. For example, it is possible that synchronized inhibition is required to prevent low-threshold mechanoreceptor inputs from driving pain circuits, and that this is lost following nerve injury, resulting in augmented DH projection neuron responses and nocifensive behavioral responses to tactile stimuli. Tests of these and other models will require probing changes in responses, including synchronous responses, across DH projection neuron populations after nerve injury; this will be challenging, however, because superficial and deep DH projection neurons are relatively few in number and are heterogeneous in both tuning properties and genetic identity.^21,23,24,28,79,80^ Future goals will be to assess responses of DH output neurons with the population measurements used here and to determine the degree to which PV^+^ interneurons and the synchronization of DH firing patterns shape projection neuron responses.

### Dysfunction of distinct dorsal horn inhibitory motifs can drive tactile over-reactivity

To address the basis for loss of population coupling following nerve injury, we manipulated three types of DH inhibitory circuit motifs and found that these alterations yielded both distinct electrophysiological changes across the DH and different behavioral manifestations. First, we silenced DH PV^+^ interneurons, which led to disorganized, desynchronized firing, predominantly in the superficial DH, and increased allodynic behaviors, mimicking the population activity alterations and behavioral over-reactivity observed following SNI and CCI. Consistent with this, *in vivo* opto-tagging experiments showed that, following SNI, PV^+^ interneurons exhibit decreased evoked firing. It is likely that most PV^+^ neurons recorded in the opto-tagged dataset are inhibitory due to their abundance,^28,32,50^ increased soma size compared to excitatory counterparts^32^ (which allows for easier signal detection via extracellular recordings), and the similarity of firing properties of control PV^+^ neurons to prior *in vivo* recordings from PV^+^ interneurons in the DH,^21^ thereby suggesting that evoked activity in inhibitory PV^+^ interneurons is reduced after nerve injury. Together, these findings suggest that alterations in PV^+^ interneurons underlie the uncoordinated population activity in the DH following nerve injury.

It is interesting that alterations in PV^+^ interneuron activity in the cortex have also been linked to changes in neuronal activity correlations and spike timing,^51–56^ indicating that PV^+^ interneurons in at least two CNS regions coordinate the precise temporal dynamics of circuit function. We suspect that this curious parallel reflects the need for fast-spiking interneurons to coordinate synchrony across CNS regions, and that calcium binding proteins such as PV are expressed in fast-spiking neurons to buffer high levels of free, ionized calcium. It is also interesting to speculate about anatomical similarities of PV^+^ interneurons across regions and across interneuron subtypes. Future work comparing anatomical features, like axonal arborization patterns, across DH interneurons could provide insight into how PV^+^ neurons organize temporal processing across large areas, and comparisons could be made between cortical PV^+^ interneurons and other cortical inhibitory subtypes. Nevertheless, since SNI causes a reduction in touch-evoked excitation of DH PV^+^ interneurons *in vivo* and intrinsic excitability and homeostatic plasticity measured *in vitro*,^45^ future studies to investigate the basis of their altered physiological properties are needed.

Interestingly, inhibitory PV^+^ interneurons form two types of inhibitory synapses in the DH: axo-dendritic synapses, contributing to FFI, and axo-axonic synapses, contributing to GABA_A_R-dependent PSI^28,45,74^ (Figure S7F). Silencing all GABA_A_R-dependent PSI does not physiologically replicate the altered network activity following nerve injury (Figures 5F–5H) suggesting that silencing axo-axonic PV^+^ terminals would not be sufficient to drive the temporal disorganization observed after nerve injury and in PV^+^ silencing. Thus, either PV^+^ interneuron axo-dendritic connections must be compromised or both axo-axonic and axo-dendritic connections must be compromised to cause the alterations in temporal correlations in SNI and PV^+^ interneuron-silenced mice. However, the PV^+^ silencing strategy used does not exclude excitatory neurons, and therefore, it is also possible that the silencing of excitatory PV^+^ interneurons contributes to network phenotypes. These findings thus point to a role for PV^+^ DH interneurons in coordinated network activity in the DH.

Finally, it is noteworthy that while nerve injury and silencing PV^+^ interneurons caused reduced synchronous firing in the DH and concomitant mechanical allodynia, but little to no change in overall physiological sensitivity across the DH as a whole, silencing either GABA_A_R-dependent PSI or Rorβ-mediated FFI led to *increased* sensitivity across the DH and *enhanced* synchronous firing but not mechanical allodynia. It is worth noting that all models of DH inhibition disruption are developmental silencing strategies and result in increased reactivity or firing in the DH; however, only silencing PV^+^ interneurons results in decreased temporal spiking precision and correlated activity patterns, which mimic what is observed following nerve injury. Thus, opposing changes in spiking precision and correlated activity are associated with distinct behaviors, further implicating temporally decorrelated activity, and not general over-reactivity of DH interneurons, as the culprit driving mechanical allodynia following nerve injury. This study focuses on the role PV^+^ interneurons play in organizing DH temporal network activity and how alterations in PV^+^ activity may contribute to pain states, but it is unlikely that PV^+^ interneurons are the only disrupted interneuron population contributing to the development of neuropathic pain, and more studies using *in vivo* recordings, subtype-specific interneuron targeting, and additional population-level analyses are needed to gain a more complete view of circuit dysfunction. Our findings, which emphasize the importance of temporal encoding of touch signals in the DH, lead us to suggest that approaches that reinstate normal patterns of synchronous firing across DH interneurons may help to restore normal behavioral reactivity to tactile stimuli and perception following nerve injury.

### Limitations of the study

Using *in vivo* MEA recordings in anesthetized mice, we investigated how sensitivity, tuning, spike timing, and network dynamics in the spinal cord DH change following peripheral nerve injury and ensuing mechanical allodynia. The anesthetized state is a limitation of the study, and it will be important in future research to investigate these features in awake, behaving animals. Additionally, we used developmental inactivation strategies to silence distinct DH circuit motifs, and future studies should be done to acutely manipulate these circuits and to monitor DH network activity and pain behaviors in behaving mice.

## STAR★METHODS

Detailed methods are provided in the online version of this paper and include the following:

### RESOURCE AVAILABILTY

#### Lead contact

Further information and requests for resources and reagents should be directed to and will be fulfilled by the lead contact, David Ginty (david_ginty@hms.harvard.edu).

#### Materials availability

This study did not generate new unique reagents.

#### Data and code availability

- All data reported in this study will be shared by the lead contact upon request.
- This paper does not report original code
- Any additional information required to reanalyze the data reported in this paper is available from the lead contact upon request.

### EXPERIMENTAL MODEL AND SUBJECT DETAILS

All mice were handled and housed in accordance with the Harvard Medical School Institutional Animal Care and Use Committee. A mix of genetic backgrounds (C57BL/6J, CD1, 129S1/SvImJ) and female and male mice were used in this study. Animals were group housed with littermates on a 12-h light/dark cycle. Tail biopsies and/or ear notching tissue samples were used for genotyping.

### METHOD DETAILS

#### Spared nerve injury

The spared nerve injury (SNI) model of neuropathic pain was used to induce mechanical allodynia in mice. Mice were anesthetized using 2% isoflurane and an incision over the biceps femoris muscle on the lateral thigh was made to expose the sciatic nerve. The peroneal and tibial branches of the sciatic nerve were ligated and transected while sparing the sural branch.^60^ Sham surgeries involved exposure of the sciatic nerve without ligation and transection. Behavioral tests for allodynia scoring were performed at Day 0 prior to SNI surgery, Day 7 post-surgery, and Day 28 post-surgery (for animals in the chronic SNI condition).

#### Chronic constriction injury

The chronic constriction injury (CCI) model of neuropathic pain was used to induce mechanical allodynia in mice. Mice were anesthetized using 2% isoflurane and an incision over the biceps femoris muscle on the lateral thigh was made to expose the sciatic nerve. The sciatic nerve was ligated four times above the peroneal, tibial, and sural branch.^87^ Sham surgeries involved exposure of the sciatic nerve without ligation. Behavioral tests for allodynia scoring were performed at Day 0 prior to CCI surgery, Day 7 post-surgery.

#### *In vivo* spinal cord multielectrode array (MEA) recordings

Recordings were amplified, filtered (0.1–7.5 kHz bandpass), and digitized (20 kHz) using a headstage amplifier and recording controller (Intan Technologies RHD2132 and Recording Controller). Data acquisition was controlled with open-source software (Intan Technologies Recording Controller version 2.07).

*In vivo* recordings were performed on animals between 6 and 24 weeks of age. Animals were administered dexamethasone 1 to 2 h before recording and anesthetized using urethane (1 mg/kg, Sigma). Temperature of the animal was monitored and maintained (TC-344B, Warner Instruments) between 35°C and 37.5°C using a thermoelectric heater (C3200-6145, Honeywell) embedded in castable cement (Aremco). Surgery was performed to expose the spinal cord. An incision was made above T13 to L6 of the spine and the surrounding tissue was removed exposing the spinal column. The vertebrae between L4 and L5 were then teased apart to expose the dorsal spinal cord. The spine was then stabilized using custom clamps to prevent movement. The dura was removed from atop the spinal cord and a 32-channel silicon probe (Cambridge Neurotech ASSY-37 H4 with 200 core fiber attached-for opto-tagging) was inserted into the lateral hindpaw region of the dorsal horn.

To confirm probe placement, the hindpaw was gently brushed while monitoring multiple channels for evoked spikes. If the receptive field was not on the lateral hindpaw the probe was removed and reinserted in a new location. Recordings began 20 min after probe insertion. A 0.2-mm diameter, Teflon-tipped indenting probe was controlled by a dual-mode force controller (Aurora Scientific 300C-I) and used to indent the lateral hindpaw. The position, force, and displacement of the indenter were commanded with custom MATLAB (version 2019a) scripts controlling a Nidaq board (National Instruments, NI USB 6259). Force steps were applied atop the minimum force required to keep the indenting probe in contact with the skin. The lateral hindpaw was stimulated with the indenting probe at a minimum of two locations which were manually determined to be receptive field hotspots for the majority of simultaneously recorded units.

#### Spike sorting

Open-source software (JRCLUST version 3.2.5) was used to automatically sort action potentials into clusters, manually refine clusters, and classify clusters as single or multi-units.^86^ The voltage traces were filtered with a differentiation filter of order 3. Frequency outliers were removed with a threshold of 10 median absolute deviations (MADs). Action potentials were detected with a threshold of 4.5 times the standard deviation of the noise. Action potentials with similar times across sites were merged and action potentials were then sorted into clusters with a density-based-clustering algorithm (clustering by fast search and find of density peaks) with cutoffs for log_10_(r) at −3 and log_10_(d) at 0.6. Clusters with a waveform correlation greater than 0.99 were automatically merged. Outlier spikes (>6.5 MADs) were removed from each cluster.

Manual cluster curation was performed with JRCLUST split and merge tools to ensure single unit isolation. Clusters were classified as putative single units if waveforms were large with respect to baseline, a clear refractory period in the cross-correlogram (interspike intervals > 1ms) was observed, and if they were clearly distinct and separable from neighboring clusters. Spike times for single units were exported and processed in Python (3.8.5).

#### Local field potentials

Local field potentials (LFPs) were examined during spontaneous and evoked activity periods. Voltage waveforms from each electrode site were low-pass filtered at 250 Hz with an 8-pole Butterworth filter to produce LFP waveforms. Power spectral densities (PSDs) of LFP waveforms were computed using Welch’s method in the Python module Scipy.Signal. PSDs across electrode sites were averaged and relative gamma power was calculated by measuring the ratio of power within bands of interest (30–80 Hz) to total power in the power spectrum.

#### Extracellular waveform characteristics

K-means clustering was performed using waveform statistics including trough-to-peak ratio, waveform slope, and trough-to-peak duration. The somatosensory cortex (S1) waveforms and control spinal cord waveforms analyzed here were from units recorded in previously published datasets.^21,68^ K = 3 was chosen to clearly separate waveforms in S1 (similar to the separation of visual cortex waveforms previously observed^69^) into two regular spiking groups and one fast-spiking group. To compare spinal cord and S1 waveforms, we used K = 3 revealing less separable groups in spinal cord waveforms. No differences in extracellular waveforms were observed between sham and SNI spinal cord neurons.

#### Optical stimulation and identification of spinal cord neurons

We used an optical tagging strategy to identify genetically defined dorsal horn interneuron populations. Interneurons expressing excitatory opsins were opto-tagged by delivering pulses (1–20ms) of blue light (4–10 mW/mm^2^ at fiber tip) to the surface of the spinal cord through an optical fiber (200 μm core diameter; NA = 0.66) attached to Cambridge Neurotech ASSY-37 H4 optrodes. Light was delivered from a 470 nm LED (M470F3, Thorlabs). Optical stimulation was performed after mechanical stimulation. At the conclusion of each experiment, 25μL of 5mM NBQX (5 mM, Tocris, dissolved in H_2_O) was applied to the surface of the spinal cord to block possible recurrent glutamatergic transmission. Abolished tactile responses to steps of indentation on the hindpaw determined efficient block of glutamatergic transmission (normally between 10 and 20 min after NBQX application, Figures S3A and S3B) and optical stimulation was repeated. Neurons that responded to stimulation both before and after NBQX application are determined to be opto-tagged.^21^ A modified stimulus-associated spike latency test (SALT^68,88^) was additionally used to confirm short light-evoked spike latencies (<10ms, Figure S3C) and low spike jitter in opto-tagged units.

#### Indentation and brush response properties

Tactile responsive single units were identified by responding to 500 ms steps of indentation at varying innocuous forces, between 1 and 75 mN. We subdivided step indentations into 3 different time periods to monitor different aspects of neuronal responses: ON response: 0–50 ms after stimulus onset; OFF response: 0–50 ms after stimulus offset; and Sustained response: 0–200 ms before stimulus offset. Thresholds for all units were determined by bootstrapping the baseline firing rate 1000 times to generate 95% confidence intervals and detecting the smallest stimulus within the ON/OFF/Sustained response windows that exceeds the upper bound.

Units that had no response threshold (only baseline firing detected) were excluded. Baseline firing was computed over a 1.5s period prior to the indentation stimuli. Peristimulus time histograms (PSTHs) were generated to show the average response across all units within a condition, genotype, and/or that have been optically tagged. These PSTHs were created with 10 ms time bins unless otherwise noted.

The lateral hindpaw was lightly stroked with a soft 1.2mm wide brush for 9 min (the brush and amount of time stroking the paw are consistent with the behavioral dynamic brush assay). For each neuron responding above baseline to brush stimuli, maximum evoked firing rates were computed for each minute of brush stimulation and these values were then averaged across all brush sessions.

#### Firing correlation analyses

Signal, noise, and synchrony cross-correlations were calculated between pairs of simultaneously recorded neurons. To calculate signal correlations, spiking responses to various stimuli were averaged across trials and the Pearson correlation coefficient of mean responses (PSTHs, 50 ms bins) between pairs of neurons were computed. Trial-by-trial spike count correlations were used to determine noise correlations. Cross-correlograms were generated (using Python module Scipy.Signal) from spiking data in 1 ms bins to determine paired firing synchrony at 0 time lag.

Population coupling was calculated as follows.^61^ Simultaneously recorded single unit activity was summed into a population rate with 1 ms resolution. The population rate was used to compute a spike-triggered population rate for each unit (not including the spikes of that unit). To compare between recordings, conditions, and genotypes each spike-triggered population rate was normalized by subtracting the median spike-triggered population rate of shuffled spiking data (randomized spike times) for each experiment. These values reflect normalized synchronous firing at a population level and are plotted as normalized population firing rates.

#### Indentation latencies and jitter measurements

Latency and jitter of single units were calculated in response to 10 mN and 75 mN step indentations of the skin. The distribution of first spike latencies to each force step was compared to a shuffled distribution (shuffled at least 100 times). The time when the distribution exceeded the 95% confidence interval of the shuffled distribution was determined to be the latency. The standard deviation of the first spike latencies across trials was then calculated to determine the jitter. A minimum of 50 trials were used to calculate latencies and jitter.

#### Spinal cord immunohistochemistry of free-floating sections

Adult mice were anesthetized with isoflurane and perfused with 10mL of 1X Phosphate Buffer Saline (PBS), followed by 20 mL of 4% paraformaldehyde (PFA) in PBS at room temperature. Vertebral columns were dissected and were post-fixed in 4% PFA at 4°C for 24 h. Lumbar spinal cord coronal sections (60 μm) were cut on a vibrating blade microtome (Leica VT100S) and processed for immunohistochemistry.^28,74^ Tissue samples were rinsed in 50% ethanol/water solution for 30 min to allow for enhanced antibody penetration followed by three washes in high salt PBS each lasting 10 min. The tissue was then incubated with primary antibodies (goat anti-mCherry (1:1000, AB0040, Scigen), rabbit anti-GFP (1:1000, A-11122, Thermo Fisher Scientific)) in high salt PBS containing 0.3% Triton X-100 (HS PBSt) for 48 h at 4°C. The tissue was washed in HS PBSt, then incubated in a secondary antibody solution in HS PBSt overnight at 4°C. Secondary antibodies included species-specific Alexa Fluor 488 and 546 conjugated IgGs (1:500; Life Technologies), and IB4 (1:500; Alexa 647 conjugated, L21411, Molecular Probes). Tissue sections were then mounted on glass slides, coverslipped with Fluoromount Aqueous Mounting Medium (Sigma) and stored at 4°C.

#### Dynamic brush assay

Dynamic mechanical allodynia was determined as follows.^48^ Hypersensitivity was measured by stroking the lateral side of the injured or sham hindpaw from heel to toe with a soft 1.2mm wide brush. Behaviors were scored from 0 to 3. No movement or a very fast lifting of the stimulated paw for less than 1 s scored as a 0. After nerve injury several pain-suggestive responses can be observed, such as sustained lifting (2 s or more) of the stimulated paw (scored as 1); lateral kicking/flinching of stimulated hindpaw (scored as 2); and licking of the stimulated paw (scored as 3). Stroking was performed for 3-min periods and repeated three times. The highest score per period was then averaged for each mouse. Sham mice scored an average score of ~0 seven days post SNI/CCI surgery and SNI/CCI mice scored ~2.3/2.5 respectively. Efficient induction of mechanical allodynia was determined by a score >1.5.

### QUANTIFICATION AND STATISTICAL ANALYSIS

Statistical tests were conducted using the SciPy stats module (Python 3.8.5) or GraphPad Prism. Both non-parametric tests and parametric tests were used, depending on data normality, for comparing two independent groups (Mann-Whitney U test or unpaired t test), and multiple groups (Kruskal-Wallis test/one-way ANOVA or two-way ANOVA for multiple groups with multiple timepoints). All post-hoc comparisons performed are indicated in the figure legends. Only significantly different p values are reported and a p < 0.05 was considered significant. All error bars plotted display 95% confidence intervals (CI) unless otherwise noted. All box and whisker plots show median, lower and upper quartiles, and minimum to maximum values. Additional details on sample sizes and statistical tests for each experiment can be found in the figure legends, main text, and supplemental table.

## Supplementary Material

1

## Figures and Tables

**KEY RESOURCES TABLE:** 

REAGENT or RESOURCE	SOURCE	IDENTIFIER

Antibodies		

Goat polyclonal anti-mCherry	Sicgen	Cat# AB0040, RRID:AB_2333093
Rabbit polyclonal anti-GFP	Thermo Fisher Scientific	Cat# A-11122, RRID:AB_221569
IB4 (Alexa 647 conjugated)	Molecular Probes	Cat# L21411, RRID:AB_2314665
Donkey anti-Goat IgG Alexa Fluor 546	Thermo Fisher Scientific	Cat# A-11056, RRID:AB_2534103
Donkey anti-Rabbit IgG Alexa Fluor 488	Thermo Fisher Scientific	Cat# A-21206, RRID:AB_2535792

Chemicals, peptides, and recombinant proteins		

Urethane	Sigma	Cat# U2500
NBQX disodium salt	Tocris	Cat# 1044
Paraformaldehyde, reagent grade, crystalline	Millipore Sigma	Cat# P6148-500G

Experimental models: Organisms/strains		

Mouse: Vgat^iresCre^	The Jackson Laboratory	RRID:IMSR_JAX:016,962
Mouse: CCK^iresCre^	The Jackson Laboratory	RRID:IMSR_JAX:012,706
Mouse: Lbx1^FlpO^	Bourane et al.^81^	N/A
Mouse: Advil^Cre^	Hasegawa et al.^82^	RRID:IMSR_JAX:032,536
Mouse: PV^Cre^	Hippenmeyer et al.^83^	RRID:IMSR_JAX:017320
Mouse: Rorβ^iresCre^	The Jackson Laboratory	RRID:IMSR_JAX:023,526
Mouse: R26^LSL-ChR2-YFP^	Madisen et al.^84^	RRID:IMSR_JAX:012,569
Mouse: Gabrb3^flox^	The Jackson Laboratory	RRID:IMSR_JAX:008,310
Mouse: Vgat^flox^	The Jackson Laboratory	RRID:IMSR_JAX:012,897
Mouse: RC::PFtox	Kim et al.^73^	N/A
Mouse: Vgat-ChR2-EYFP	The Jackson Laboratory	RRID:IMSR_JAX:014548
Mouse: PV-tdTomato	Kaiser et al.^85^	MGI: 97821

Software and algorithms		

JRCLUST	Jun et al.^86^	https://github.com/JaneliaSciComp/JRCLUST
MATLAB	Mathworks	https://www.mathworks.com/products/MATLAB.html; RRID: SCR_001622
Python	Van Rossum and Drake (1995)	https://www.python.org/

Other		

Multielectrode arrays	Cambridge Neurotech	ASSY-37H4
RHD USB interface board Intan Technologies C3100	Intan Technologies	Part #C3100
Winsor & Newton Cotman Watercolor Brush - Designers’ Round, Short Handle, Size 0	Blick	Item #:05039-1000

## Highlights

- Nerve injury does not induce general over-reactivity in spinal cord interneurons
- Spike timing and neural activity correlations are disrupted following nerve injury
- PV^+^ interneurons exhibit reduced firing after nerve injury *in vivo*
- Silencing PV^+^ interneurons disrupts spike timing and activity correlations
