# Supplementary material for: Nerve injury disrupts temporal processing in the spinal cord dorsal horn through alterations in PV+ interneurons
Source: Cell Rep. Author manuscript; Available in PMC 2024 May 18. (PMC11101906; doi:10.1016/j.celrep.2024.113718)
Supplement: 1 [file NIHMS1990866-supplement-1.pdf]

Cell Reports, Volume *43*

Supplemental information

Nerve injury disrupts temporal processing in the spinal cord dorsal horn through alterations in PV^+ interneurons

Genelle Rankin, Anda M. Chirila, Alan J. Emanuel, Zihe Zhang, Clifford J. Woolf, Jan Drugowitsch, and David D. Ginty

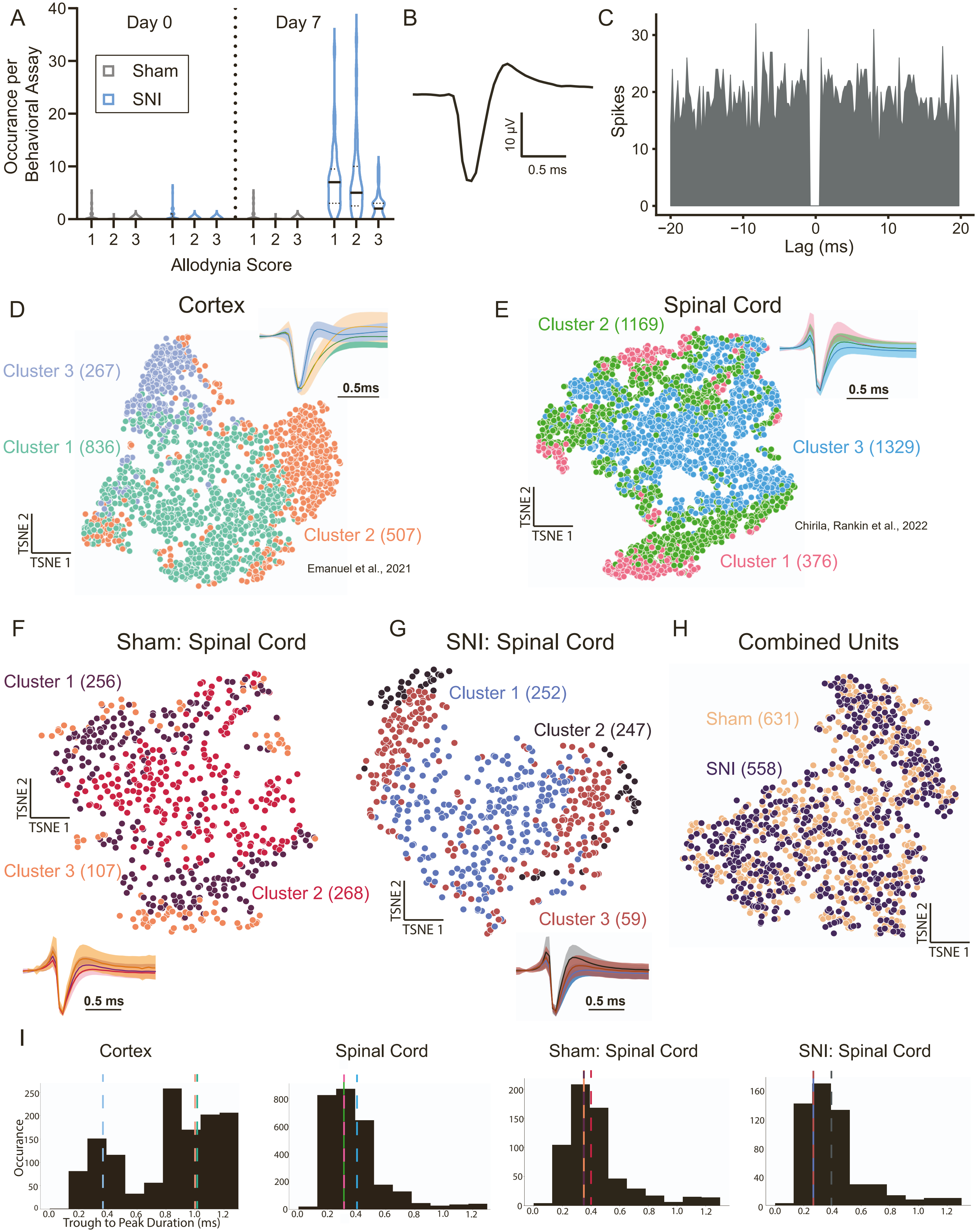
A
Day 0
Day 7
Sham
SNI
Occurance per Behavioral Assay
Allodynia Score
B
10 µV
0.5 ms
C
Spikes
Lag (ms)
D
Cortex
Cluster 3 (267)
Cluster 1 (836)
Cluster 2 (507)
0.5ms
TSNE 2
TSNE 1
Emanuel et al., 2021
E
Spinal Cord
Cluster 2 (1169)
Cluster 3 (1329)
Cluster 1 (376)
0.5 ms
Chirila, Rankin et al., 2022
F
Sham: Spinal Cord
Cluster 1 (256)
Cluster 3 (107)
Cluster 2 (268)
G
SNI: Spinal Cord
Cluster 1 (252)
Cluster 2 (247)
Cluster 3 (59)
H
Combined Units
Sham (631)
SNI (558)
I
Cortex
Spinal Cord
Sham: Spinal Cord
SNI: Spinal Cord
Occurance
Trough to Peak Duration (ms)

Figure S1

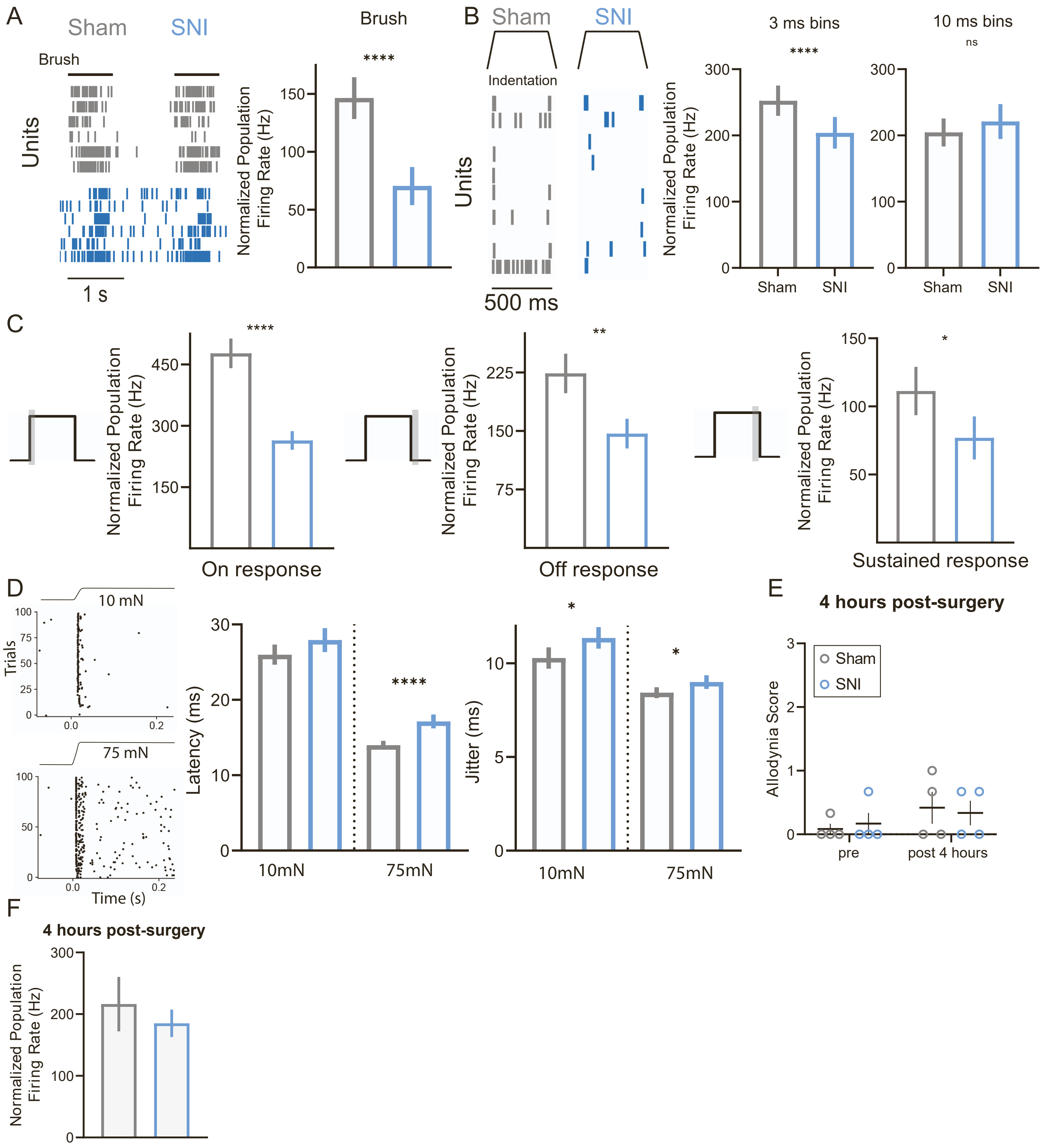
A
Sham
SNI
Brush
Units
1 s
Brush
****
Normalized Population Firing Rate (Hz)
150
100
50
0
B
Sham
SNI
Indentation
Units
500 ms
3 ms bins
****
10 ms bins
ns
Normalized Population Firing Rate (Hz)
300
200
100
0
Sham
SNI
C
Normalized Population Firing Rate (Hz)
450
300
150
****
On response
225
150
75
**
Off response
150
100
50
*
Sustained response
D
10 mN
75 mN
Trials
100
75
50
25
0
0.0
0.2
Time (s)
Latency (ms)
30
20
10
0
****
10mN
75mN
Jitter (ms)
10
5
0
*
*
E
4 hours post-surgery
Sham
SNI
Allodynia Score
3
2
1
0
pre
post 4 hours
F
4 hours post-surgery
Normalized Population Firing Rate (Hz)
300
200
100
0

Figure S2

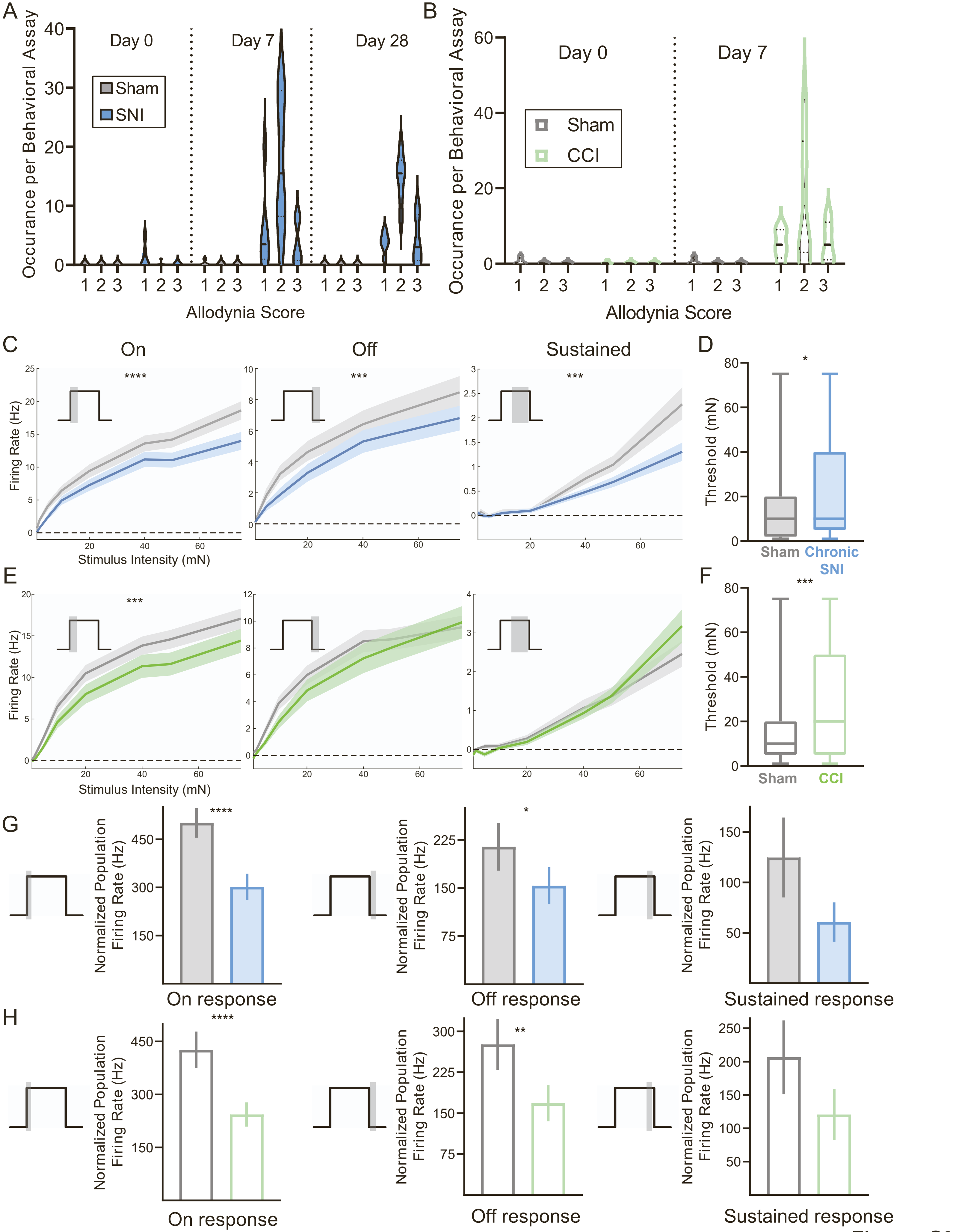
A
Occurance per Behavioral Assay
Day 0
Day 7
Day 28
Sham
SNI
Allodynia Score
B
Occurance per Behavioral Assay
Day 0
Day 7
Sham
CCI
Allodynia Score
C
On
Off
Sustained
Firing Rate (Hz)
Stimulus Intensity (mN)
****
***
***
D
Threshold (mN)
*
Sham
Chronic SNI
E
Firing Rate (Hz)
Stimulus Intensity (mN)
***
F
Threshold (mN)
***
Sham
CCI
G
Normalized Population Firing Rate (Hz)
****
*
On response
Off response
Sustained response
H
Normalized Population Firing Rate (Hz)
****
**
On response
Off response
Sustained response

Figure S3

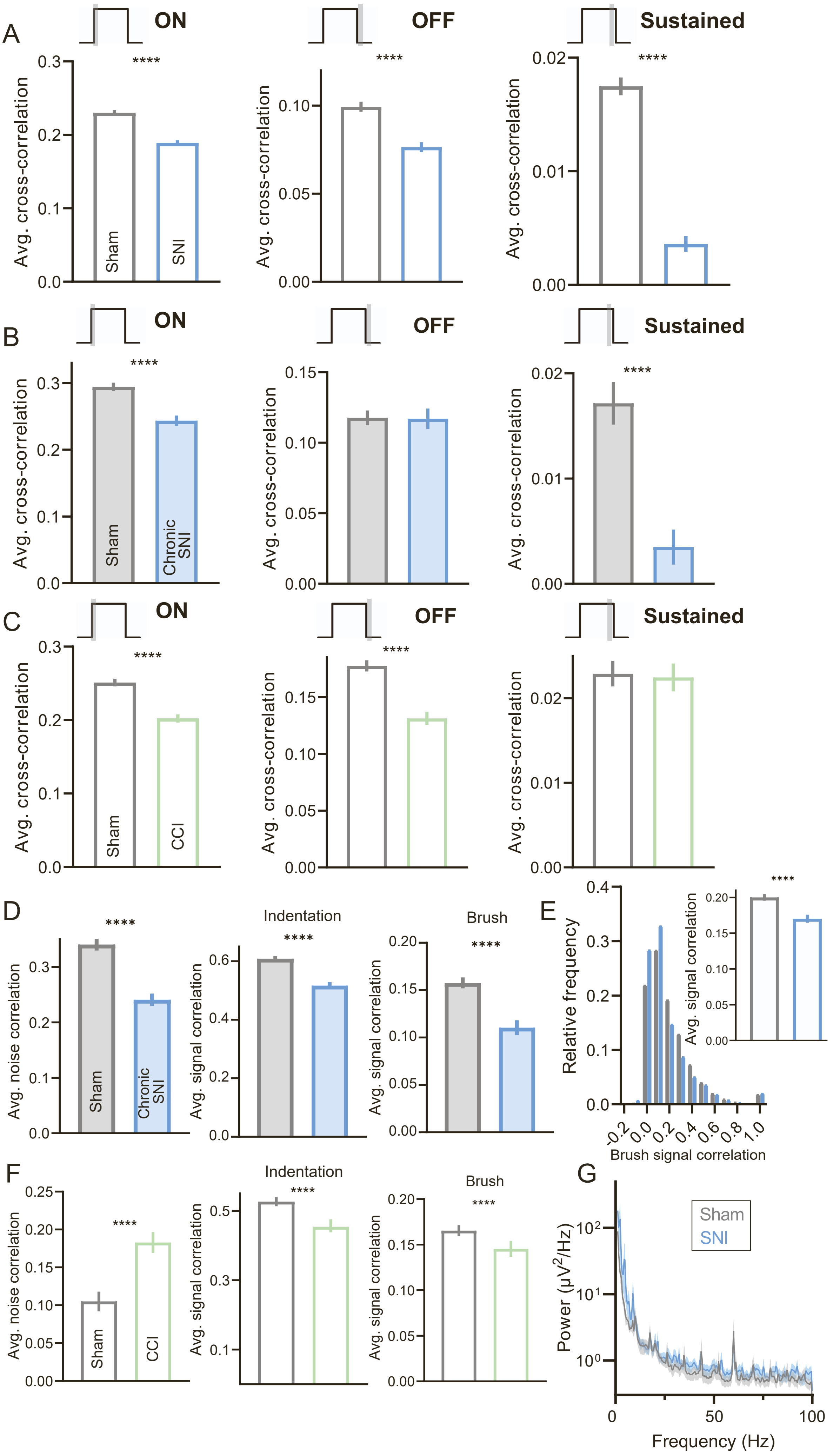
A
ON
OFF
Sustained
Avg. cross-correlation
****
Sham
SNI
B
Chronic SNI
C
CCI
D
Avg. noise correlation
Avg. signal correlation
Indentation
Brush
E
Relative frequency
Brush signal correlation
F
G
Sham
SNI
Power (μV²/Hz)
Frequency (Hz)

Figure S4

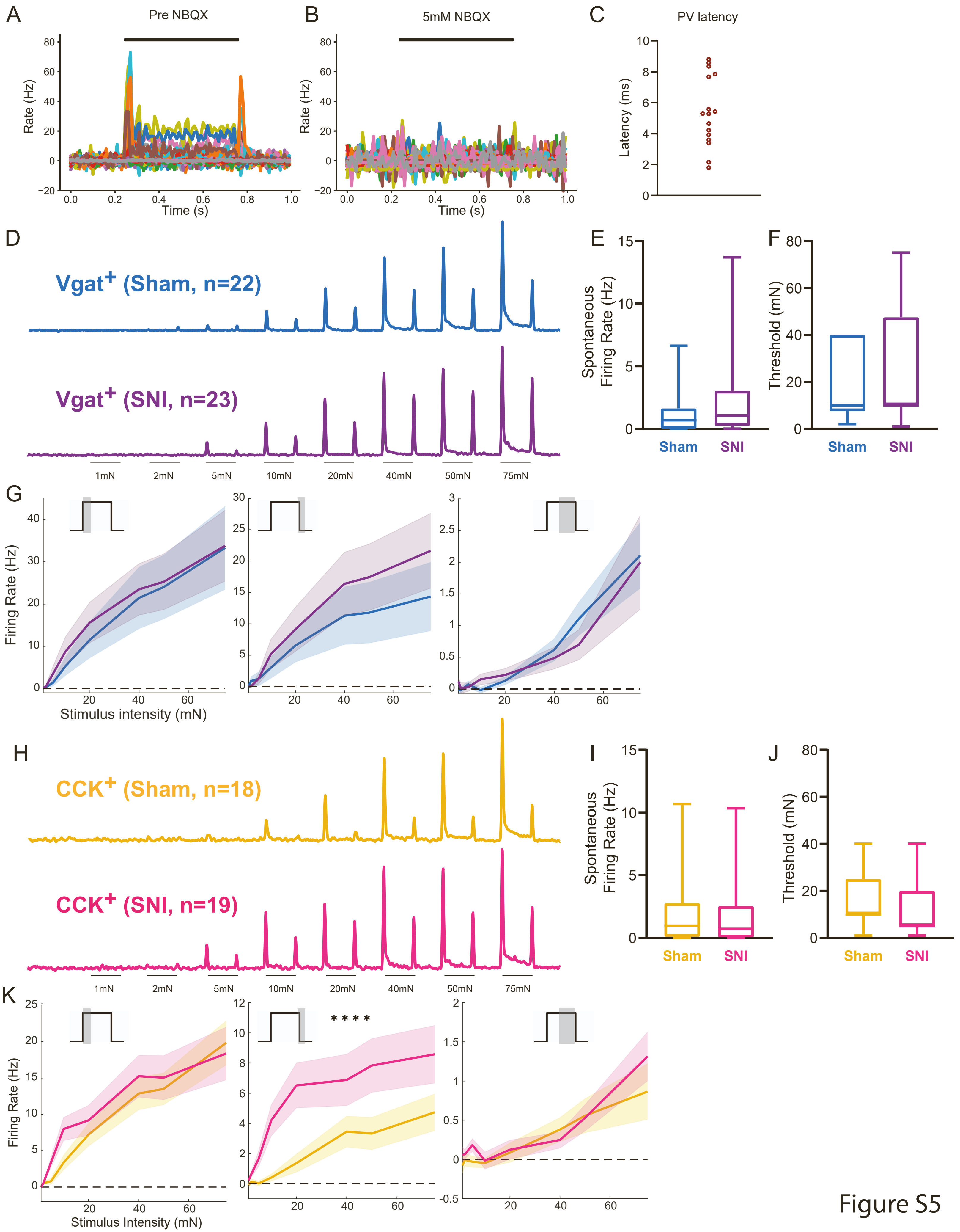

A
Pre NBQX
Rate (Hz)
Time (s)
B
5mM NBQX
C
PV latency
Latency (ms)
D
Vgat+ (Sham, n=22)
Vgat+ (SNI, n=23)
1mN
2mN
5mN
10mN
20mN
40mN
50mN
75mN
E
Spontaneous Firing Rate (Hz)
Sham
SNI
F
Threshold (mN)
G
Firing Rate (Hz)
Stimulus intensity (mN)
H
CCK+ (Sham, n=18)
CCK+ (SNI, n=19)
I
J
K
* * * *
Stimulus Intensity (mN)

Figure S5

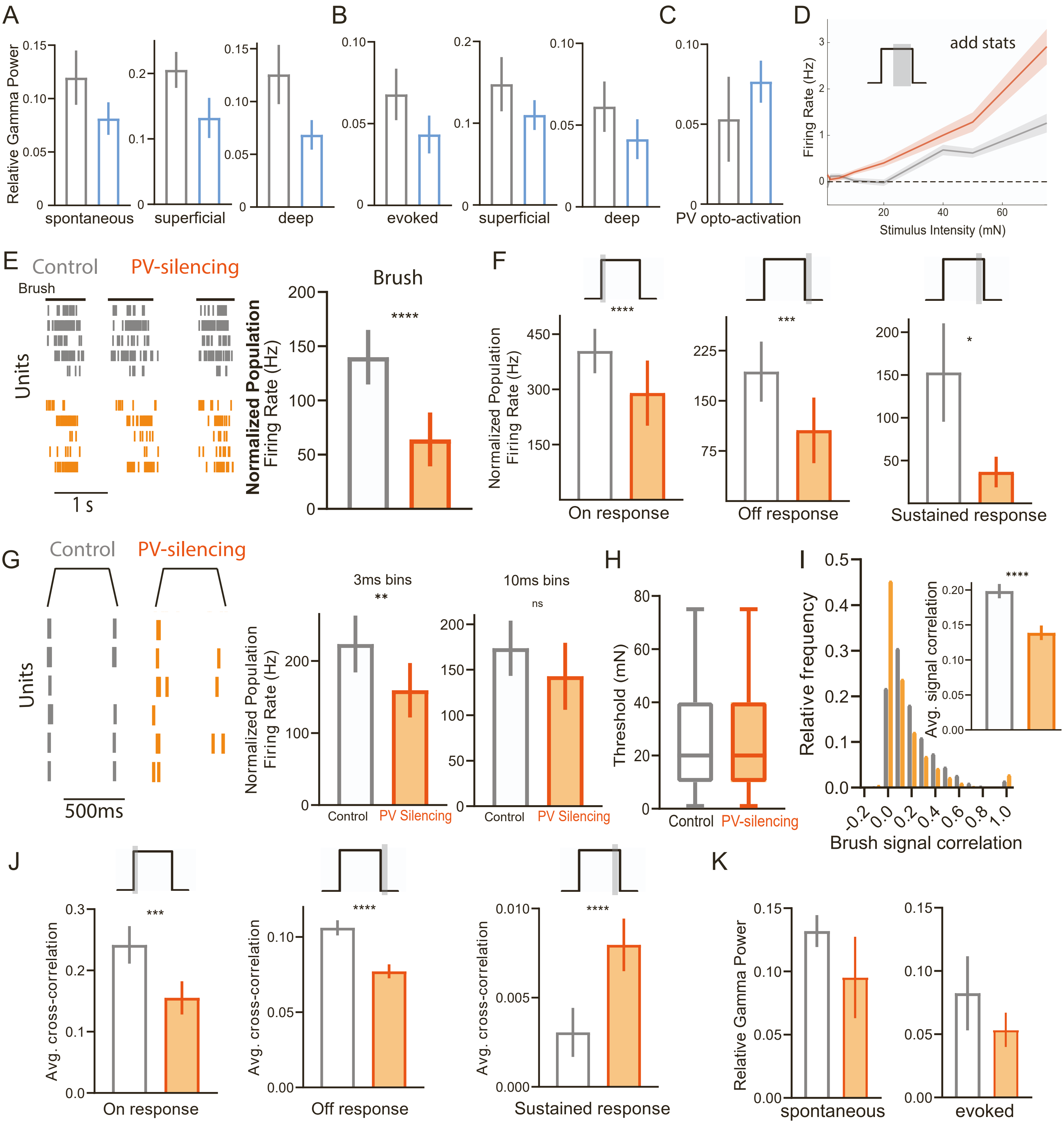
A
Relative Gamma Power
spontaneous
superficial
deep
B
evoked
superficial
deep
C
PV opto-activation
D
add stats
Firing Rate (Hz)
Stimulus Intensity (mN)
E
Control
PV-silencing
Brush
Units
1 s
Brush
****
Normalized Population
Firing Rate (Hz)
F
****
***
*
Normalized Population
Firing Rate (Hz)
On response
Off response
Sustained response
G
Control
PV-silencing
Units
500ms
3ms bins
**
10ms bins
ns
Normalized Population
Firing Rate (Hz)
Control
PV Silencing
Control
PV Silencing
H
Threshold (mN)
Control
PV-silencing
I
****
Avg. signal correlation
Relative frequency
Brush signal correlation
J
***
****
****
Avg. cross-correlation
Avg. cross-correlation
Avg. cross-correlation
On response
Off response
Sustained response
K
Relative Gamma Power
spontaneous
evoked

Figure S6

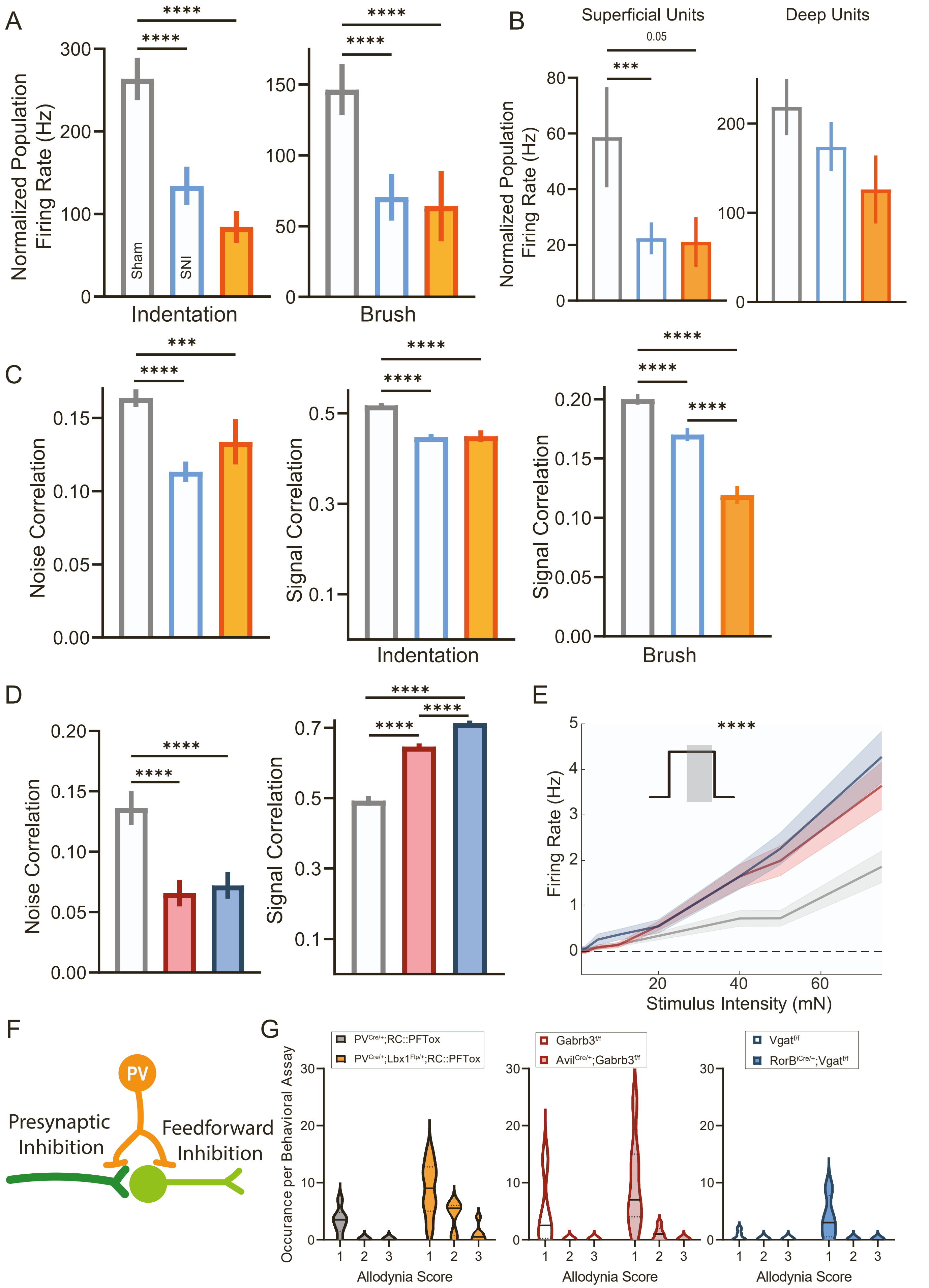
A
Normalized Population Firing Rate (Hz)
Sham
SNI
Indentation
Brush
B
Superficial Units
Deep Units
0.05
Normalized Population Firing Rate (Hz)
C
Noise Correlation
Signal Correlation
Indentation
Brush
D
Noise Correlation
Signal Correlation
E
Firing Rate (Hz)
Stimulus Intensity (mN)
F
PV
Presynaptic Inhibition
Feedforward Inhibition
G
$PV^{Cre/+}$;RC::PFTox
$PV^{Cre/+}$;$Lbx1^{Flp/+}$;RC::PFTox
$Gabrb3^{f/f}$
$Avil^{Cre/+}$;$Gabrb3^{f/f}$
$Vgat^{f/f}$
$RorB^{iCre/+}$;$Vgat^{f/f}$
Occurance per Behavioral Assay
Allodynia Score

Figure S7

Supplemental figure legends

Figure S1. Allodynic behaviors and extracellular waveform features of spinal cord interneurons, related to Figure 1 and Figure 4

A. Behaviors exhibited over dynamic brush assay per allodynia score category in sham (N=22) and SNI (N=19) mice. 1: sustained lifting (2 sec or more) of the stimulated paw. 2: lateral kicking/ flinching of stimulated hindpaw. 3: licking of the stimulated paw.

B. Example extracellular waveform from a DH neuron.

C. Corresponding auto-correlogram for the neuron shown in Figure S1B.

D. t-SNE visualization of somatosensory cortex extracellular waveforms clustered using k-means clustering (n= 1610 units; see Methods). As previously reported[69], three primary waveform subtypes emerged: two regular-spiking (green, cluster 1; orange, cluster 2) and one fast-spiking (light blue, cluster 3). Normalized mean waveforms in different clusters are shown ±SD.

E. Extracellular waveforms for DH units (n = 2874 units) clustered as in (D). Unlike cortex, DH waveforms are not clearly separated into three groups (pink, cluster 1; green, cluster 2; blue, cluster 3).

F. Sham DH unit extracellular waveforms (n = 631 units) clustered as in D (dark purple, cluster 1; red, cluster 2; orange, cluster 3).

G. SNI DH unit extracellular waveforms (n = 558 units) clustered as in D (blue, cluster 1; black, cluster 2; brown, cluster 3).

H. Combined sham and SNI unit waveforms do not cluster separately.

I. Trough-to-peak durations for each region/condition. Cortical unit waveforms have a bimodal distribution, highlighting the duration difference between fast-spiking (dashed purple line, cluster 3; median: 0.35 ms) and regular-spiking (dashed green line, cluster 1; median: 1.05 ms; dashed orange line, cluster 2; median: 1.00 ms) units. Spinal cord unit waveforms have a unimodal distribution (medians for cluster per cluster plotted as dashed lines).

Figure S2. Temporal organization of DH firing across milliseconds to seconds and for hours to weeks after SNI surgery, related to Figure 2

A. Raster plots (left) showing temporal alignment of simultaneously recorded units responding to skin stroke in sham and SNI conditions followed by population coupling quantified as normalized population firing rate (right). Mann-Whitney *U* test.
B. Raster plots showing temporal alignment of simultaneously recorded units responding to 50 mN indentation steps (left), population coupling with expanded 3 ms time bins (middle) and population coupling with expanded 10 ms time bins (right). Mann-Whitney *U* tests.
C. Population coupling during 50 ms periods of evoked activity: On (50 ms from the start of the indentation step; left), Off (50 ms from the end of the indentation step; middle), and Sustained (50 ms prior to the end of the indentation step; right). Mann-Whitney *U* test.
D. Left: raster plots of one sham neuron responding to the onset of 10 mN and 75 mN indentation steps across trials. Right: quantification of latency and jitter to indentation steps across neurons.
E. Dynamic allodynia score compared prior to surgery and 4 hours post-surgery in sham (N=4) and SNI (N=4) mice. Error bars: SEM
F. Population coupling 4 hours post nerve injury. Mann-Whitney *U* test.
Bars: mean. Error bars: 95% CI. Mann-Whitney *U* tests, *$p < 0.05$, **$p < 0.01$, ****$p < 0.0001$. See Table S1 for statistic details.

Figure S3. Allodynic behaviors and neuronal firing patterns in chronic SNI and CCI models of mechanical allodynia, related to Figure 2
A. Behaviors exhibited over dynamic brush assay per allodynia score category in sham (N=4) and chronic SNI (N=6) mice. 1: sustained lifting (2 sec or more) of the stimulated paw. 2: lateral kicking/ flinching of stimulated hindpaw. 3: licking of the stimulated paw.
B. As in (A), for sham (N=5) and CCI (N=5) mice.
C. Average baseline-subtracted firing rates (±SEM) for DH units in sham and chronic SNI groups at step indentation onset (On: 0-50 ms after step onset), offset (Off: 0-50 ms after step offset), and sustained (Sustained: 0-200 ms before step offset) periods. On: t*wo-way ANOVA* [$F(1,2176) = 25.43$, $p < 0.0001$]. Off: t*wo-way ANOVA* [$F(1,2176) = 14.22$, $p = 0.0002$]. Sustained: t*wo-way ANOVA* [$F(1,2176) = 13.49$, $p = 0.0002$].
D. Distribution of indentation thresholds across DH neurons in sham (n=135, gray) and chronic SNI (n=124, blue) mice. Mann-Whitney *U* test.

E. Average baseline-subtracted firing rates (±SEM) for DH units in sham and CCI groups at step indentation onset (On: 0-50 ms after step onset), offset (Off: 0-50 ms after step offset), and sustained (Sustained: 0-200 ms before step offset) periods. On: t*wo-way ANOVA* [F (1,2216) = 15.09, p = 0.0001].

F. Distribution of indentation thresholds across DH neurons in sham (n=157, gray) and CCI (n=113, green) mice. Mann-Whitney *U* test.

G. Population coupling during 50 ms periods of evoked activity: On (50 ms from the start of the indentation step; left), Off (50 ms from the end of the indentation step; middle), and Sustained (50 ms prior to the end of the indentation step; right) in sham and chronic SNI mice. Mann-Whitney *U* test.

H. As in (G), for sham and CCI mice.

Bars: mean. Error bars: 95% CI. Number of animals/ cells (N). *p < 0.05, **p < 0.01, ***p < 0.001, ****p < 0.0001. See Table S1 for experimental details.

Figure S4. Dorsal horn neuron firing correlations in models of mechanical allodynia, related to Figure 3

A. Synchrony cross-correlations for neuron pairs during 50 ms periods of evoked activity: On (50 ms from the start of the indentation step; left), Off (50 ms from the end of the indentation step; middle), and Sustained (50 ms prior to the end of the indentation step; right) in sham and SNI mice.

B. As in (A), for sham and chronic SNI mice.

C. As in (A), for sham and CCI mice.

D. Noise (left) and signal correlations (indentation: middle, brush: right) for neuron pairs after chronic SNI.

E. Distribution of brush signal correlations for pairs of DH neurons in sham and SNI mice. Insets: average signal correlations.

F. As in (D), for neuron pairs after CCI.

G. Average LFP (local field potential) power spectra during periods of spontaneous activity in sham (N=8) and SNI (N=7) mice.

Bars: mean. Error bars: 95% CI. Mann-Whitney *U* tests, *p < 0.05, **p < 0.01, ****p < 0.0001. See Table S1 for statistic details.

Figure S5. Characterizing Vgat$^+$ and CCK$^+$ interneuron activity following SNI, related to Figure 4

A. Simultaneously recorded units responding to 50 mN steps of indentation (black bar).

B. Indentation responses to 50 mN step indentations (black bar) abolished by glutamatergic block using NBQX applied to the surface of the spinal cord.

C. Average latency to first spike after optical stimulation for PV$^+$ DH interneurons in the sham condition.

D. Mean baseline-subtracted firing rate PSTHs for sham and SNI Vgat$^+$ neurons.

E. Indentation thresholds across Vgat$^+$ neurons in sham (N=4, n=22) and SNI (N=5, n=23) mice.

F. Spontaneous firing rates of Vgat$^+$ interneurons.

G. Average baseline-subtracted firing rates (±SEM) for Vgat$^+$ neurons in sham and SNI groups at step indentation On (left), Off (middle), and Sustained (right) periods.

H. Mean baseline-subtracted firing rate PSTHs for sham and SNI CCK$^+$ neurons.

I. Indentation thresholds across CCK$^+$ neurons in Sham (N=4, n=18) and SNI (N=6, n=19) mice.

J. Spontaneous firing rates of CCK$^+$ interneurons.

K. Average baseline-subtracted firing rates (±SEM) for CCK$^+$ neurons in sham and SNI groups at step indentation On, Off, and Sustained periods. Off: t*wo-way ANOVA* [F (1,280) = 29.77, p < 0.0001].

Bars: mean. Error bars: 95% CI. Number of animals/ cells (N/n). *p < 0.05, **p < 0.01, ***p < 0.001.

Figure S6. Allodynic behaviors and dorsal horn neuronal firing patterns in the PV-silencing condition, related to Figure 5

A. Average relative LFP power within gamma frequency bands (30-80 Hz) during periods of spontaneous activity across all electrodes (left), superficial electrodes (corresponding to units in Figure 2B; middle), and deep electrodes (corresponding to units in Figure 2B; right). Error bars: SEM

B. As in (A), for evoked activity.

C. Average relative power within gamma frequency bands (30-80 Hz) during optogenetic activation of PV^+ neurons. Error bars: SEM

D. Average baseline-subtracted firing rates (±SEM) for dorsal horn neurons in control (grey) and PV-silencing (orange) groups at sustained periods of indentation. Sustained: t*wo-way ANOVA* [F (1,1664) = 19.49, p < 0.0001].

E. Raster plots showing temporal alignment of simultaneously recorded units responding to strokes (right) in sham and SNI conditions followed by population coupling quantified as normalized population firing rate. Mann-Whitney *U* test.

F. Population coupling during 50 ms periods of evoked activity: On (50 ms from the start of the indentation step; left), Off (50 ms from the end of the indentation step; middle), and Sustained (50 ms prior to the end of the indentation step; right) in control and PV-silencing mice. Mann-Whitney *U* test.

G. Raster plots showing temporal alignment of simultaneously recorded units responding to 50 mN indentation steps (left), population coupling with expanded 3 ms time bins (middle) and population coupling with expanded 10 ms time bins (right). Mann-Whitney U tests.

H. Indentation thresholds between control (N=4) and PV-silencing mice (N=4). Mann-Whitney *U* test.

I. Distribution of brush signal correlations for pairs of DH neurons. Insets: average signal correlations. Mann-Whitney *U* test.

J. As in F, for synchrony cross-correlations.

K. Average relative LFP power within gamma frequency bands (30-80 Hz) during periods of spontaneous (top) and evoked (bottom) activity across all electrodes. Error bars: SEM

Bars: mean. Error bars: 95% CI. Number of animals (N). *p < 0.05, **p < 0.01, ***p < 0.001, ****p < 0.0001.

Figure S7. Altered correlated activity is comparable between PV-silencing and SNI conditions, related to Figure 5

A. Population coupling to both indentation (left) and brush (right) stimuli comparing a randomly shuffled subset of the sham and SNI datasets with the PV-silencing dataset. Indentation: One-way ANOVA with post-hoc Tukey's test [F (2, 353) = 18.91; p < 0.0001]. Brush: One-way ANOVA with post-hoc Tukey's test [F (2, 371) = 12.39; p < 0.0001].

B. Superficial (left) and deep (right) population coupling comparing a shuffled subset of the sham and SNI datasets with the PV-silencing dataset. Superficial: One-way ANOVA with post-hoc Tukey's test [$F (2, 81) = 6.703$; $p = 0.0020$].

C. Noise (left) and signal correlations (indentation: middle, brush: right) for neuron pairs from a randomly shuffled subset of the sham and SNI datasets with the PV-silencing dataset. Noise: One-way ANOVA with post-hoc Tukey's test [$F (2, 4184) = 8.124$; $p = 0.0003$]. Signal (indentation): One-way ANOVA with post-hoc Tukey's test [$F (2, 4184) = 8.124$; $p = 0.0003$].

D. Noise and signal correlations (indentation) for neuron pairs. Noise: Kruskal-Wallis H test with post-hoc Dunn's test ($H[2,6099] = 60.27$; $p < 0.0001$). Signal: Kruskal-Wallis H test with post-hoc Dunn's test ($H[2, 6099] = 618.2$; $p < 0.0001$).

E. Average baseline-subtracted firing rates (±SEM) for dorsal horn neurons in control, PSI KO (red), and FFI KO (blue) groups at Sustained periods of indentation. Sustained: t*wo-way ANOVA* [$F (2,2640) = 32.49$, $p < 0.0001$].

F. Diagram of presynaptic and feedforward inhibitory synapses formed by PV^+ DH neurons.

G. Behaviors exhibited over dynamic brush assay per allodynia score category in control (*PV^{Cre};RC::PFtox*; N=6) and PV-silencing (*PV^{Cre};$Lbx1^{FlpO}$;RC::PFtox*; N=6) mice, control (*$Gabrb3^{f/f}$*; N=4) and PSI KOs (*$Avil^{Cre}$;$Gabrb3^{f/f}$*; N=6) mice, and control (*$Vgat^{f/f}$*; N=5) and FFI KOs (*$Ror\beta^{iCre}$;$Vgat^{f/f}$*; N=4). 1: sustained lifting (2 sec or more) of the stimulated paw. 2: lateral kicking/ flinching of stimulated hindpaw. 3: licking of the stimulated paw.

Bars: mean. Error bars: 95% CI. Number of animals/ cells (N). *$p < 0.05$, **$p < 0.01$, ***$p < 0.001$. See Table S1 for experimental details.

Table S1

N=animals n=pairs of cells

Figure 2	panel	sub-panel	Sham (N/n)	Condition (N/n)
				**SNI**
#	**B**	indentation	22/642	19/479
#	**C**	superficial	21/120	18/102
		deep	21/485	18/364
				**Chronic SNI**
#	**F**	indentation	4/103	5/116
#	**G**	superficial	4/ 43	5/ 31
		deep	4/ 109	5/ 112
				**CCI**
#	**H**		4/ 166	4/ 127
#	**I**	superficial	4/ 47	4/ 29
		deep	4/ 119	4/ 98

Figure S2	panel	sub-panel	Sham (N/n)	SNI (N/n)
#	**A**		15/318	12/321
#	**B**	3ms	22/642	19/479
		10ms	22/642	19/479
#	**C**	on	22/642	19/479
		off	22/642	19/479
		sus	22/642	19/479
#	**D**	10mN		
		latency	21/179	18/151
		jitter	21/179	18/151
		75mN		
		latency	21/447	18/318
		jitter	21/447	18/318
	**E**		4	4
#	**F**		4/120	4/ 87

Figure 3	panel	sub-panel	Sham (N/n)	Condition (N/n)
				**SNI**
	**B**		21/9351	19/7535
				**Chronic SNI**
			4/ 2959	5 /2041
				**CCI**
			4/ 4863	4/ 3938
	**D**		22/9921	17/7008
	**F**		21/9351	19/7535

Figure S3	panel	sub-panel	Sham (N/n)	Condition (N/n)
				**Chronic SNI**
#	**D**		4/135	5/124
				**CCI**
#	**F**		4/ 157	4/ 113
				**Chronic SNI**
#	**G**	on	4/103	5/116
		off	4/103	5/116
		sus	4/103	5/116
				**CCI**
	**H**	on	4/ 166	4/ 127
		off	4/ 166	4/ 127
		sus	4/ 166	4/ 127

Figure S4	panel	sub-panel	Sham (N/n)	Condition (N/n)
				**SNI**
	**A**	on	21/9351	19/7535
		off	21/9351	19/7535
		sus	21/9351	19/7535
				**Chronic SNI**
	**B**	on	4/ 2959	5 /2041
		off	4/ 2959	5 /2041
		sus	4/ 2959	5 /2041
				**CCI**
	**C**	on	4/ 4863	4/ 3938
		off	4/ 4863	4/ 3938
		sus	4/ 4863	4/ 3938
				**Chronic SNI**
	**D**	noise	4/ 2959	5 /2041
		indentation	4/ 2959	5 /2041
		brush	4 /4281	5/ 2296
				**SNI**
	**E**		15/5040	12 /4840
				**CCI**
	**F**	noise	4/ 2050	4/ 1794
		indentation	4/ 3844	4/ 2010
		brush	4/ 3724	4/ 2022

n= number of cells

Figure 5	panel	sub-panel	Control (N/n)	Condition (N/n)	Condition (N/n)
				**PV-silencing**	
#	**A**	indentation	4/ 99	4/108	
#	**B**	superficial	4/ 21	4 /24	
		deep	4/ 78	4 /84	
	**C**		4/1410	4/1384	
	**D**	noise	4/1410	4/1384	
		indentation	4/1410	4/1384	
				**PSI KO**	**Rorβ KO**
#	**F**		3/ 94	3/114	3/107
	**G**		3/1558	3/ 2453	3/ 2088
#	**H**		3/ 94	3/114	3/107

Figure S6	panel	sub-panel	Control (N/n)	Condition (N/n)
			**Sham**	**SNI**
	**A**		8	7
		superficial	8	7
		deep	8	7
	**B**		8	7
		superficial	8	7
		deep	8	7
	**C**		3	3
				**PV-silencing**
#	**E**	brush	4/ 99	4/127
#	**F**	on	4/ 99	4/108
		off	4/ 99	4/108
		sus	4/ 99	4/108
#	**G**		4/ 99	4/108
			4/ 99	4/108
	**H**		4/ 99	4/108
	**I**		4/1410	4/1458
	**J**	on	4/1410	4/1384
		off	4/1410	4/1384
		sus	4/1410	4/1384
	**K**	spontaneous	3	4
		evoked	3	4

Figure S7	panel	sub-panel	Control (N/n)	Condition (N/n)	Condition (N/n)
			**Sham shuffled (n)**	**SNI shuffled (n)**	**PV-silencing (N=4)**
#	**A**	indentation	125	125	108
		brush	125	125	127
#	**B**	superficial	30	30	24
		deep	100	100	84
	**C**	noise	1400	1400	1387
		indentation	1800	1800	1797
			**Control**	**PSI KO**	**Rorβ KO**
	**D**	noise	3/1558	3/ 2453	3/ 2088
		indentation	3/1558	3/ 2453	3/ 2088

Figure	panel	test	statistic	p-value
1	D	Mann-Whitney U	109679	0.006
2	B	Mann-Whitney U	114728	<0.0001
	C superficial	Mann-Whitney U	4565	0.0016
	F	Mann-Whitney U	3256	<0.0001
	G superficial	Mann-Whitney U	204	<0.0001
	H	Mann-Whitney U	8570	0.0024
	I superficial	Mann-Whitney U	394	0.0018
3	B SNI	Mann-Whitney U	30879621	<0.0001
	B Chronic SNI	Mann-Whitney U	2103818	<0.0001
	B CCI	Mann-Whitney U	8055216	<0.0001
	D	Mann-Whitney U	31474258	<0.0001
	F	Mann-Whitney U	30076950	<0.0001
4	G	Mann-Whitney U	76	0.0322
	H	Unpaired t test	t=2.705, df=	0.0113
5	A	Mann-Whitney U	2533	<0.0001
	B superficial	Mann-Whitney U	135	0.0071
	C	Mann-Whitney U	765752	<0.0001
	D noise	Mann-Whitney U	913091	0.0024
	D signal	Mann-Whitney U	789032	<0.0001
	I	Mann-Whitney U	0.5	0.0043
S2	A	Mann-Whitney U	33004	<0.0001
	B 3ms	Mann-Whitney U	106197	<0.0001
	C on	Mann-Whitney U	126765	<0.0001
	C off	Mann-Whitney U	156318	0.0024
	C sus	Mann-Whitney U	156449	0.0372
	D latency 75mN	Mann-Whitney U	57131	<0.0001
	D jitter 10mN	Mann-Whitney U	11532	0.0217
	D jitter 75mN	Mann-Whitney U	68612	0.0226
S3	D	Mann-Whitney U	6961	0.0236
	F	Mann-Whitney U	6477	0.0001
	G on	Mann-Whitney U	5931	<0.0001
	G off	Mann-Whitney U	8963	0.0184
	H on	Mann-Whitney U	7327	<0.0001
	H off	Mann-Whitney U	8638	0.0051
S4	A on	Mann-Whitney U	30155795	<0.0001
	A off	Mann-Whitney U	31717092	<0.0001
	A sus	Mann-Whitney U	29809502	<0.0001
	B on	Mann-Whitney U	2403478	<0.0001
	B sus	Mann-Whitney U	2242082	<0.0001
	C on	Mann-Whitney U	7909746	<0.0001
	C off	Mann-Whitney U	7119617	<0.0001
	D noise	Mann-Whitney U	2447068	<0.0001
	D indentation	Mann-Whitney U	2503596	<0.0001
	D brush	Mann-Whitney U	3687353	<0.0001
	E	Mann-Whitney U	15222303	<0.0001
	F noise	Mann-Whitney U	1521367	<0.0001
	F indentation	Mann-Whitney U	3481975	<0.0001
	F brush	Mann-Whitney U	3131596	<0.0001
S6	E	Mann-Whitney U	3887	<0.0001
	F on	Mann-Whitney U	3810	<0.0001
	F off	Mann-Whitney U	4001	0.0003
	F sus	Mann-Whitney U	4343	0.0358
	G 3ms	Mann-Whitney U	4222	0.0009
	I	Mann-Whitney U	733833	<0.0001
	J on	Mann-Whitney U	3804	0.0001
	J off	Mann-Whitney U	869281	<0.0001
	J sus	Mann-Whitney U	907256	0.0171

*all tests are two-tailed

Supplemental Table

Table S1: Statistical analyses and additional details, related to Figures 1, 2, 3, 4, 5, S2, S3, S4, S6, S7
